# Trade‐Offs and Synergies Between Climate Change Mitigation, Biodiversity Preservation, and Agro‐Economic Development Across Future Land‐Use Scenarios in Brazil

**DOI:** 10.1111/gcb.70418

**Published:** 2025-08-07

**Authors:** Thomas M. R. Gérard, Sietze J. Norder, Judith A. Verstegen, Jonathan C. Doelman, Stefan C. Dekker, Floor van der Hilst

**Affiliations:** ^1^ Copernicus Institute of Sustainable Development Utrecht University Utrecht the Netherlands; ^2^ Department of Human Geography and Spatial Planning Utrecht University Utrecht the Netherlands; ^3^ PBL Netherlands Environmental Assessment Agency Den Haag the Netherlands; ^4^ NIOO‐KNAW, the Netherlands Institute of Ecology Wageningen the Netherlands

**Keywords:** agricultural expansion, carbon sequestration, deforestation, environmental impacts, GHG emissions, land use change, species richness

## Abstract

Land‐use change is a major driver of biodiversity loss and a key contributor to GHG emissions, making sustainable land use essential for biodiversity preservation and climate change mitigation. The impacts of land use change are location‐specific, shaped by the biophysical context. Consequently, the extent and nature of these impacts are deeply influenced by the spatial configuration of land‐use change. This is particularly relevant for Brazil, a global agricultural powerhouse, where agricultural expansion impacts biodiversity‐rich and carbon‐rich biomes. Understanding the future land‐use trade‐offs and synergies between agro‐economic development, biodiversity preservation, and climate change mitigation is crucial to support sustainable land use in Brazil. In this study, we quantified these trade‐offs and synergies for three SSP‐based land‐use change scenarios projected for 2050. For each scenario, we assessed the spatial variation in impacts on carbon stocks, mammal distributions, and agricultural revenues. Our results show that the agricultural economy is projected to grow at the expense of biodiversity preservation and climate change mitigation objectives, and vice versa. These trade‐offs and synergies result from changes in natural vegetation and agricultural land, driven by shifting demand for agricultural products. In particular, under the SSP3‐7.0 scenario, rising agricultural demand between 2015 and 2050 is projected to drive agricultural expansion into natural areas, increasing annual agricultural revenue by 36.5 billion USD_2015_ but reducing carbon stock by 4.5 Gt and mammal distribution areas by 3.4%. In contrast, the SSP1‐1.9 scenario projects a decline in agricultural demand over the same period, driving the conversion of agricultural land to natural vegetation. This shift increases carbon stocks by 5.6 Gt and expands mammal distribution areas by 6.8%, although it would lower annual agricultural revenue by 33.4 billion USD_2015_. Our findings further highlight opportunities to reduce trade‐offs by containing agriculture outside biodiversity‐rich and carbon‐rich biomes, in combination with strategic restoration of these regions.

## Graphical abstract

Agricultural expansion is a major driver of biodiversity loss and a key source of greenhouse gas emissions in Brazil. In this study, we quantify the impacts of future land‐use change on carbon stocks, mammal species richness, and agricultural revenue under three land‐use scenarios. Our results show that the agricultural economy is projected to grow at the expense of biodiversity preservation and climate change mitigation, and vice versa. However, our findings further highlight opportunities to reduce these trade‐offs by containing agriculture outside biodiversity‐rich and carbon‐rich areas, in combination with strategic restoration of these regions.

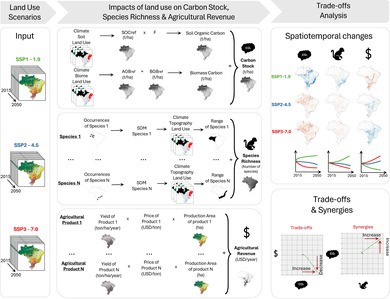

## 1 Introduction

Safeguarding biodiversity and mitigating climate change are two main challenges of the 21st century (Richardson et al. 2023). The loss of biodiversity and the impacts of climate change pose significant threats to both ecosystems and human society, including food and water insecurity and increased vulnerability to natural hazards (IPBES 2019; IPCC 2023b). In response to these threats, countries have pledged to various international agreements, such as the Convention on Biological Diversity, the Paris Agreement, and the Sustainable Development Goals (SDGs). Meeting these commitments, however, requires overcoming significant challenges related to land use and, in particular, to agricultural expansion.

Historically, the increasing demand for resources has driven extensive conversion of natural land into agricultural land (de Vries 2012; Foley et al. 2005; UNCCD 2017), making land‐use change a major driver of biodiversity loss (Díaz et al. 2019; Maxwell et al. 2016) and a key contributor to greenhouse gas emissions (Friedlingstein et al. 2023; IPCC 2022; UNCCD 2017). Therefore, to achieve their environmental and climate commitments, countries must rethink their land resource allocation and management. The core challenge lies in designing land use strategies that consider multiple objectives, including agro‐economic development, biodiversity preservation, and climate change mitigation. Ideally, land‐use strategies result in synergies by contributing to multiple objectives simultaneously. However, pursuing one objective might undermine another, resulting in trade‐offs. Assessing the cost of achieving one objective over another, as well as identifying land‐use strategies in which multiple objectives can be met concurrently, is critical to enabling governments to make informed decisions and to support sustainable development (Kennedy et al. 2016; Silva, de Castro Victoria, et al. 2023).

Such assessments are particularly relevant for Brazil, a country that has undergone profound land‐use transformations over the past 50 years, emerging as a global agricultural powerhouse (Dias et al. 2016; Stabile et al. 2020). Extensive areas of natural land have been converted into pastures, croplands, and forest plantations, while urban areas and road networks have also expanded considerably (Lapola et al. 2023; Souza et al. 2020). Nowadays, Brazil is one of the world's leading exporters of soybeans, beef, coffee, and sugar, with these exports contributing significantly to its economy (IBGE 2021; OECD and FAO 2024). However, the corresponding land‐use changes have also occurred in regions important for biodiversity preservation and terrestrial carbon storage. Brazil is home to around 13% of the world's species and encompasses some of the most important biodiversity hotspots (IPBES 2019; Joly et al. 2019; Jung et al. 2021). The expansion of agriculture in these areas represents a serious threat to biodiversity (Kehoe et al. 2017). Moreover, agriculture and land‐use change are the primary sources of Brazil's greenhouse gas emissions (MCTI 2020b). In particular, deforestation in the Amazon rainforest has significantly contributed to climate change (Brienen et al. 2015; IPCC 2022, 2023a). Understanding future land‐use trade‐offs and synergies between agro‐economic development, biodiversity preservation, and climate change mitigation is crucial to support sustainable land use in Brazil.

Previous studies in Brazil have assessed these trade‐offs and synergies by comparing different land‐use configurations. For example, Chaplin‐Kramer et al. (2015) highlighted that, in Mato Grosso, greenhouse gas emissions and biodiversity loss depend on the spatial pattern of agricultural expansion, with the magnitude of trade‐offs varying accordingly. In the same region, Silva, deCastro Victoria, et al. (2023) found that strict compliance with current forest restoration policies would result in significant trade‐offs between biodiversity preservation and climate change mitigation with agro‐economic development. They also demonstrated that trade‐offs can be reduced by the restoration of native vegetation and ecosystems. Similarly, Kennedy et al. (2016) presented a land‐use configuration in which preserving specific natural areas can sustain biodiversity while simultaneously increasing agricultural profits. National‐scale research suggests that intensifying the management of existing pastures could increase agricultural production while avoiding deforestation and associated greenhouse gas emissions, but it does not consider the impact on biodiversity (Cohn et al. 2014; Frank et al. 2017; Strassburg et al. 2014; van der Hilst et al. 2018).

The above‐mentioned studies provide meaningful insights into synergies and trade‐offs resulting from land‐use strategies and spatial planning, but they either do not cover the entire country or do not consider biodiversity preservation, climate change mitigation, and agro‐economic development simultaneously. Moreover, they do not account for the diverse potential future global change context, which would significantly influence land‐use demands. As a result, there is a lack of comprehensive national‐scale information on the trade‐offs and synergies between biodiversity preservation, climate change mitigation, and agro‐economic development. Furthermore, there is a need to understand how these trade‐offs and synergies may evolve under different future global change scenarios. In this aspiration, our research aims to quantify the potential future land‐use trade‐offs and synergies between biodiversity preservation, climate change mitigation, and agro‐economic development in Brazil by 2050. In addition, we seek to identify the land‐use dynamics driving these trade‐offs and synergies. By focusing on these trade‐offs and synergies, the research aims to provide insights into pathways for harmonizing agro‐economic development with biodiversity preservation and climate change mitigation in Brazil, paving the way for integrated solutions to global challenges.

## 2 Materials and Methods

### 2.1 General Approach

**FIGURE 1:**
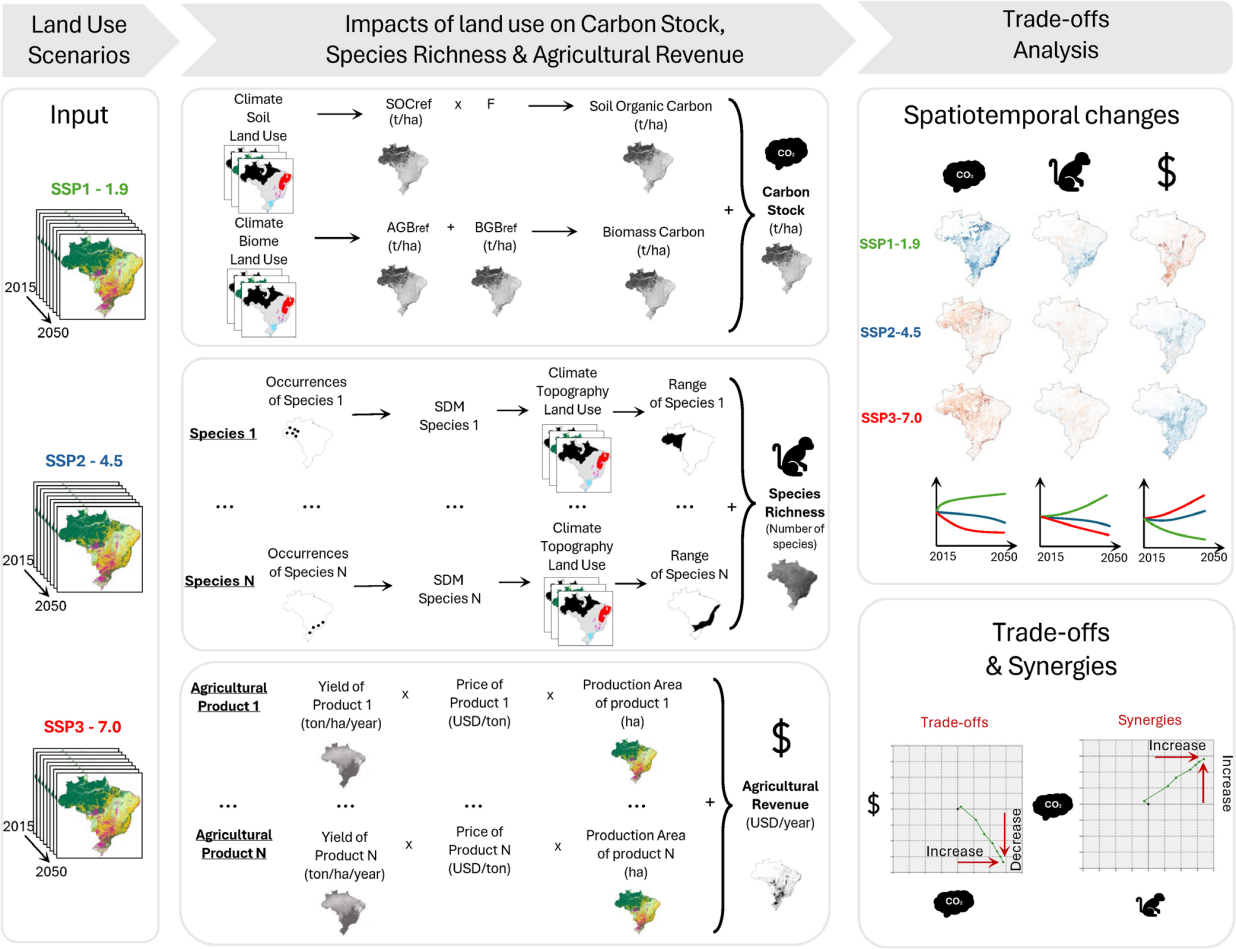
Methodological framework. The assessment of the impacts of land‐use changes on climate change mitigation, biodiversity preservation, and agro‐economic development, and a trade‐offs analysis across three scenarios. To assess the impact of land‐use on climate change mitigation, we quantify Brazil's land carbon stock, using stock change factors (F) and reference carbon stock in soil (SOCref), above‐ground biomass (AGBref), and below‐ground biomass (BGBref). To assess the impact of land‐use change on biodiversity, we quantify mammal richness by estimating the distribution of over 150 mammal species. These distributions are determined using Species Distribution Models (SDMs), one for each species. The agro‐economic assessment involves quantifying agricultural revenue by considering the revenues generated from both pasture and cropland.

This study employs land‐use projections for Brazil developed by Silva Bezerra et al. (2022), which outline three distinct land‐use scenarios towards 2050. For each scenario, projections include land‐use maps at 5‐year intervals from 2015 to 2050. For each time step, we quantify the spatial variation of three key indicators: terrestrial carbon stock, mammal species richness, and agricultural revenue. Changes in these indicators over time are used to evaluate each scenario's progress towards climate change mitigation, biodiversity preservation, and agroeconomic development. To compare projected scenario outcomes with current trends, we quantify changes in these indicators for observed land‐use change between 2015 and 2020, using data from the MapBiomas Project (2023b). Following the indicator quantification, we conduct a trade‐off and synergy analysis for each scenario. If all indicators improve simultaneously, this suggests synergies between their respective objectives. Conversely, improvements in one indicator accompanied by declines in another indicate trade‐offs. The spatiotemporal trends of each indicator and the underlying land‐use transitions offer insights into the nature of these trade‐offs and synergies. Figure 1 presents an overview of the research methodology.

Changes in terrestrial carbon stocks reflect carbon emissions or sequestration resulting from land‐use change. Currently, land‐use change impacts on carbon stocks are one of the major contributors to Brazil's greenhouse gas emissions (MCTI 2020b). However, land‐use change also represents significant potential for carbon sequestration (Heinrich et al. 2021). Accordingly, carbon stock dynamics serve as a meaningful proxy for assessing a land‐use change scenario's potential contribution to climate change mitigation. Other sources of greenhouse gas emissions, such as nitrous oxide and methane from fertilizer use and livestock, are not included in this assessment; however, they also play an important role in climate change.

Changes in mammal species richness are used as a proxy for biodiversity preservation. Mammals are selected due to their crucial roles in ecosystem functioning and services (Lacher et al. 2019) as well as the availability of comprehensive data on their spatial distribution (Marsh et al. 2022; Rondinini et al. 2011). Although species richness does not capture species composition, endemism, threat sensitivity, functional diversity, or rarity (Baldwin et al. 2017; Guerin et al. 2015; Guerin and Lowe 2015), it nonetheless provides meaningful insight into the impact of habitat loss driven by land‐use change.

Changes in agricultural revenue indicate the economic implications of land‐use change for Brazil's agricultural sector. Although production costs are not accounted for, revenue trends provide a useful proxy for evaluating economic development within the sector due to management and land‐use changes. This is especially relevant in Brazil, where agricultural growth has historically been driven by land expansion (Dias et al. 2016; Stabile et al. 2020).

This study does not account for the impacts of climate change on carbon stocks, mammal richness, or agricultural revenue. Rather, the analysis focuses exclusively on land‐use changes to isolate their impacts. Further methodological details are provided in the subsequent sections.

### 2.2 Land‐Use Scenarios

The land‐use projections used in this research were developed by Silva Bezerra et al. (2022), which model three land‐use scenarios and cover the period from 2015 to 2050. These projections provide the spatial distribution of seven land‐use categories, that is, forest, grassland, pasture, croplands, forest plantation, mosaic pattern, and “other,” at five‐year intervals, mapped as fractions at a 10 × 10‐km resolution. The projections were modeled using the LuccME (Land Use and Cover Change Modelling Environment) framework, an open‐source tool for spatially explicit land‐use change modeling developed by Brazil's National Institute for Space Research (INPE). LuccME follows a three‐step process: first, it determines the demand for each land‐use category; second, it assesses the suitability of each land‐use type based on various factors; and third, it allocates land‐use changes accordingly.

Silva Bezerra et al. (2022) ran the LuccME model for three Shared Socio‐economic Pathways (SSP) scenarios (O'Neill et al. 2014). When combined with Representative Concentration Pathways (RCP), which represent different levels of radiative forcing, the SSPs‐RCPs describe various socio‐economic and climate change contexts (van Vuuren et al. 2014). Among these, Silva Bezerra et al. (2022) selected three SSP‐RCP combinations: SSP2‐4.5 (“Middle of the road scenario”), SSP1‐1.9 (“Route toward a more sustainable world”), and SSP3‐7.0 (“Strong inequality scenario”). For each SSP‐RCP, the demand for each land use was derived from IMAGE 3.0 integrated assessment model (Stehfest et al. 2014) and subsequently used as input for LuccME. In addition to the global SSP‐RCP scenarios, Silva Bezerra et al. (2022) incorporated Brazil‐specific spatial drivers for land‐use change (e.g., proximity to roads, agricultural suitability) and national policy (e.g., implementation of the Forest Code or protected areas). These elements were directly aligned with the broader global SSPs‐RCPs narratives and were essential in determining land‐use suitability in LuccME. The initial land‐use configuration for 2015 was derived from historical land‐use maps provided by IBGE (Brazilian Institute of Geography and Statistics), based on observed land‐use patterns. The three SSP‐RCP scenarios represent land‐use projections where global and local factors are aligned.

The scenarios capture a range of potential land‐use futures, enabling a comprehensive analysis of land‐use changes under diverse socio‐economic and climate conditions. The SSP2‐4.5 scenario represents a continuation of current trends in consumption habits, with increasing demand and improved supply efficiency for agricultural products, alongside partial restrictions on agricultural expansion. In this scenario, food prices decrease, and agricultural land continues to expand (Doelman et al. 2018). In contrast, the SSP1‐1.9 scenario reflects a shift in consumption habits, with a reduced preference for animal products and a slow increase in demand for agricultural products. Simultaneously, supply efficiency improves due to higher crop yields, more productive livestock systems, and reduced food loss. In combination with a relatively lower population growth compared to SSP2‐4.5, this scenario results in decreasing food prices and a contraction of agricultural land, supported by a strict implementation of environmental and land‐use policies (Doelman et al. 2018). The SSP3‐7.0 scenario represents a world with high population growth, a relatively high preference for animal products, and rising food losses, all contributing to a strong increase in demand for agricultural products. Meanwhile, supply is less efficient, with limited improvements in crop yields and livestock system efficiency. In this context, food prices increase, and with limited land‐use regulation, agricultural land expands strongly (Doelman et al. 2018). Overall, these scenarios depict various contexts of population growth, consumption patterns, and land‐use regulations, resulting in changes in demand, supply, and prices, as well as land use (Table 1).

**TABLE 1:** Scenario narratives and associated land‐use changes.

	SSP1‐1.9	SSP2‐4.5	SSP3‐7.0
Global context			
Globalization of trade	Highly globalized	Moderately globalized	Less globalized
Crop yield	High yield improvement	Moderate yield improvement	Low yield improvement
Livestock system efficiency	High efficiency improvement	Moderate efficiency improvement	Low efficiency improvement
Animal product consumption	Reduced preference for animal products	Current preference for animal product	High preference for animal product
Price	High decline in price for agricultural product	Moderate decline in price for agricultural product	Increase in price for agricultural product
Policy context in Brazil			
Forest code	Implemented and surpassed by local restoration efforts to meet legal reserve requirements and protect areas of permanent protection	Implemented and satisfied with the trading of forest quotas between private lands with a surplus and those with a deficit of legal reserves	Forest code is not respected and control measure are discontinued
Conservation units	Fully protected	Partially protected	Not protected
Indigenous lands	Fully protected	Partially protected	Fully protected
Road network	No major federal or state roads are constructed after 2020	The ongoing paving projects are expanded, but accompanied by measures to avoid uncontrolled occupation	The ongoing paving projects are expanded and not accompanied by measures to avoid uncontrolled occupation
Land use change in Brazil (2015–2050)			
Forest	Expand by 88 million ha (+23%)	Decline by 4 million ha (−1%)	Decline by 13 million ha (−3%)
Grassland	Decline by 12 million ha (−7%)	Decline by 35 million ha (−19%)	Decline by 55 million ha (−29%)
Pasture	Decline by 58 million ha (−36%)	Expand by 20 million ha (+12%)	Expand by 42 million ha (+26%)
Cropland	Decline by 12 million ha (−15%)	Expand by 16 million ha (+21%)	Expand by 20 million ha (+26%)
Forest plantation	Decline by 5 million ha (−54%)	Decline by less than 1 million ha (−7%)	Expand by 1 million ha (+9%)
Other	Expand by less than 1 million ha (+23%)	Expand by 3 million ha (+9%)	Expand by 4 million ha (+10%)

*Note:* Adapted from Silva Bezerra et al. (2022) and Doelman et al. (2018).

The three scenarios also reflect different policy contexts resulting in distinct land‐use change dynamics within Brazil (Silva Bezerra et al. 2022). Specifically, the scenarios consider different levels of implementation of the Forest Code, and the protection of conservation units (UCs) and indigenous land (ILs), key land‐use policies aimed at preventing deforestation, mitigating climate change, and preserving biodiversity. Under the Forest Code, private landowners are required to maintain a legal reserve (LR), which is a minimum natural area that must be preserved on their land. If landowners do not comply with LR requirements, they must either restore their land or purchase forest credits from another owner who exceeds their LR obligations (Camara et al. 2023; da Silva, Millington, et al. 2023). In addition, Brazilian authorities have established UCs and ILs to prevent deforestation in sensitive areas (Camara et al. 2023). In SSP2‐4.5, the Forest Code is implemented with a compensation mechanism (i.e., forest quotas), but illegal deforestation persists in UCs and ILs, particularly in more densely populated areas. Ongoing infrastructure projects, including road paving, continue with further expansion planned by 2030 and 2040. As a result, forest cover is expected to decline by 2050, with agricultural land continuing to expand. In contrast, SSP1‐1.9 reflects a strict and efficient enforcement of UCs, ILs, and the Forest Code. Additionally, no major federal or state roads are assumed to be constructed after 2020. By 2050, this is projected to lead to a reduction in agricultural land, progressively replaced by secondary vegetation. SSP3‐7.0 projects a weak implementation of the Forest Code, with UCs no longer protected. Still, ILs benefit from strict and efficient protection. The ongoing paving projects are assumed to expand without measures to avoid uncontrolled land occupation. By 2050, this scenario results in a significant decrease in natural vegetation with a continued expansion of agricultural land. The characteristics of the scenarios are summarized in Table 1.

To facilitate the quantification of carbon stocks and agricultural revenue, the “Cropland” land‐use category of the projection of Silva Bezerra et al. (2022) was subdivided into annual, perennial, and semi‐perennial croplands. Similarly, the “Mosaic” land‐use category was divided into individual land‐use categories to ensure clear and distinct classifications. The methods used for disaggregating the “Cropland” and “Mosaic” categories are detailed in Supporting Information Sections 1.1 and 1.2, respectively.

### 2.3 Carbon Stock

The terrestrial carbon stocks, expressed in tonnes of carbon per hectare (t/ha), are calculated following the methods of the IPCC guidelines for national greenhouse gas inventories (IPCC 2006, 2019). These methods rely on reference values of carbon stocks of soil, biomass, dead wood, and litter, specific to the land‐use and environmental context (e.g., climate zone, soil type). Dead wood and litter are not included due to the lack of reference values for croplands, forest plantations, or pastures. Compared to the other components, they contain a minor portion of the carbon and are often set to zero (IPCC 2006, 2019). While the IPCC provides global default reference values for soil and biomass carbon, we use Brazil‐specific and location‐specific reference values. This allows for a spatially explicit quantification of carbon stocks across Brazil. We apply this method at each time step of the projections, assuming an instantaneous change in carbon stock following land‐use conversion. As a result, the spatiotemporal trends in carbon sequestration or emissions can be deduced for each scenario.

Biomass carbon encompasses two different pools: above‐ground biomass (AGB) and below‐ground biomass (BGB). AGB includes all living vegetation above the soil (e.g., stems, stumps, branches, bark, seeds and foliage) while BGB encompasses all living roots. In this research, we estimate biomass carbon using location‐specific reference carbon stocks of AGB and BGB. These reference values, provided by the Brazilian Ministry of Innovation and Sciences (MCTI), are specific to biomes and climates in Brazil and refer to natural vegetation cover without any human disturbance (MCTI 2020a; SIRENE et al. 2020). Therefore, these reference values are used for forests and grasslands, assuming instantaneous mature systems. For other land uses such as pasture, agriculture, and forest plantations, we use IPCC reference values specific to land use and climate conditions (IPCC 2006, 2019). For urban areas and “other” land uses, we assume no biomass carbon (IPCC 2006, 2019). A summary of the sources of the reference values is provided in the Supporting Information 2.1.

In regard to soils, we quantify the organic carbon (SOC) in both mineral soil (i.e., moderately or well‐drained soils with low levels of organic matter) and organic soil (i.e., poorly drained soils with high amounts of organic matter such as peat and muck soil). To estimate SOC, we multiply the reference SOC values by stock factor changes (F), expressing how land use, management practices, and organic inputs influence the reference carbon stock. As detailed in Supporting Information 2.2, reference SOC values specific to land use, climate, and soil conditions are calculated using the 2015 MapBiomas Solo Beta collection (MapBiomas Project 2023a). For the stock factor (F), we used land‐use and biome‐specific values provided by MCTI (MCTI 2020a; SIRENE et al. 2020). Only SOC in the top 30 cm of soil is quantified, as this layer is most affected by land‐use changes (IPCC 2006, 2019). Furthermore, soil drainage, strongly affecting the SOC of organic soil (IPCC 2006, 2019), is not considered, as organic soils represent less than 1% of the soils in Brazil (IBGE 2024).

### 2.4 Mammal Richness

To estimate changes in mammal richness, we project the distribution of mammal species at each timestep of the scenarios. The projected distributions of mammals are determined using a logistic Species Distribution Model for each species (SDMs). Although logistic models may not fully capture the complex interactions between environmental factors and species distributions due to the assumption of linear relationships (Fletcher and Fortin 2018), they are effective at handling multiple variables, making them well‐suited for this research.

The development of each logistic model requires preparing presence and absence records for each species. First, we extracted species presence coordinate records from the GBIF (GBIF 2024), SpeciesLink (CRIA 2024) and SALVE (ICMBio 2024). These online databases often contain temporal, geographic, and taxonomic inconsistencies. Therefore, we filtered the data to retain only reliable records and address geographic sampling biases (data preparation workflow is included in Supporting Information 3.1). After preparing the dataset, 234 distinct species of mammals remained, with an average of 66 presence records per species (15,342 records in total). Regarding absence records, few reliable data are available (Barbet‐Massin et al. 2012). Therefore, it is common to use “pseudo‐absence” resulting from random sampling in areas with habitats unsuitable for the species (Barbet‐Massin et al. 2012; Fletcher and Fortin 2018). This method has been shown to be the most reliable for the logistic model (Barbet‐Massin et al. 2012; Fletcher and Fortin 2018), especially when using a large number of pseudo‐absences with equal weighting for presence and absence (Barbet‐Massin et al. 2012). Applying those recommendations, 1000 absences were randomly sampled per species, using the expert species range maps of Marsh et al. (2022) to demarcate (un)suitable areas. Finally, we sampled the values of ten different environmental variables at the coordinates of each presence and absence record, including four land‐use types (forest, cropland, forest plantation, grassland‐pasture), two topographic variables (mean slope, mean elevation), and four climate variables (mean annual temperature, mean temperature seasonality, mean annual precipitation, mean precipitation seasonality). These types of variables are commonly used to describe suitable and unsuitable habitat conditions in SDMs (Martínez‐Minaya et al. 2018). A more detailed description of these variables is provided in Supporting Information 3.2.

After preparing the presence and absence data, a logistic model is calibrated for each species to generate a habitat suitability map for Brazil, which can then be converted into a distribution map by applying a threshold. The process is as follows: first, highly correlated variables are excluded to ensure independence between variables (Dormann et al. 2013). Then, the best combination of the remaining variables is selected using the Akaike Information Criterion (AIC), a statistical measure that compares the goodness of fit across different models. The model with the lowest AIC value is chosen as the optimal model (Venables and Ripley 2002). Subsequently, the selected model is applied across Brazil to estimate the probability of species occurrence in each grid cell, indicating habitat suitability. Finally, the optimal threshold for distinguishing between suitable and unsuitable habitats is determined by calculating the True Skill Statistic (TSS) for each potential threshold between 0 and 1. The TSS is a metric representing the model's overall discriminative ability (Allouche et al. 2006; Fletcher and Fortin 2018). The threshold that maximizes the TSS is selected as the optimal value, as recommended by Jiménez‐Valverde and Lobo (2007). This process is described in more detail in Supporting Information 3.3.

Model robustness, or its ability to perform consistently across different data subsets, is assessed using five‐fold cross‐validation (Hooten and Hobbs 2015). This technique involves dividing the presence and absence data into five equal folds. A logistic model is trained using four of the five folds, while the remaining fold is used to test the model's discriminative performance. This process is repeated, with five distinct fold combinations. To evaluate the discriminative performance of each model, two metrics are used: the Area Under the Curve (AUC) along with the TSS (Allouche et al. 2006; Fletcher and Fortin 2018). The AUC is a threshold‐independent metric, with values above 0.5 indicating that the model performs better than random chance. Meanwhile, the maximal TSS value, described above, indicates poor performance when below 0. Species and respective logistic models with an average AUC below 0.5 or an average TSS below 0 are excluded from further analysis. After this validation process, 151 species and models remained.

The habitat suitability and distribution of the remaining 151 species are then determined at each timestep of the land‐use projections. To isolate the effect of land‐use change on species distributions, climate and topography are assumed to remain constant over time. In addition, to reduce overprediction, we geographically constrain the projected distributions within expert range maps (Marsh et al. 2022) as recommended in the literature (Mendes et al. 2020; Velazco et al. 2020). This method ensures that the predicted distributions do not include areas far from where the species actually occur. Ultimately, we create a potential mammal richness map for each timestep of the land‐use scenarios by counting the number of species in each grid cell.

### 2.5 Agricultural Revenue

The agricultural revenue, expressed in USD_2015_ per hectare (USD_2015_/ha), is calculated spatially explicitly across Brazil at each timestep of the scenario projections, enabling the analysis of spatiotemporal trends in agricultural revenue generation. In particular, the agricultural revenue is calculated by summing the revenues generated from pastures and annual, perennial, and semi‐perennial croplands, as represented by Equation (1). Forestry revenue is excluded due to the absence of data on future prices of forestry products.

(1) $$R_{t}=R_{\mathrm{pa},t}+R_{\mathrm{ca},t}+R_{\mathrm{cp},t}+R_{\mathrm{cs},t}$$

Where *R*_*t*_ represents the revenue at time *t* (USD_2015_/year), *R*_Pa,*t*_ is the revenue from pasture at time *t* (USD_2015_/year), *R*_ca,*t*_ is the revenue from annual cropland at time *t* (USD_2015_/year), *R*_cp,*t*_ is the revenue from perennial cropland at time *t* (USD_2015_/year), *R*_cs,*t*_ is the revenue from semi‐perennial cropland at time *t* (USD_2015_/year). The revenue for each of these land uses is computed using Equation (2):

(2) $$R_{i,t}=P_{i,t}\times Y_{i,t}\times A_{i,t}$$

where *R*_*i,t*_ is the revenue for land use *i* at time t (USD_2015_/year), *P*_*i,t*_ is the price of the agricultural product of land use *i* at time *t* (USD_2015_/ton), *Y*_*i,t*_ is the yield of land use *i* at time *t* (ton/ha/year) and *A*_*i,t*_ is the area of land use *i* at time *t* (ha). The areas of each land use are provided by the land‐use projections, but additional assumptions are necessary to determine prices and yields. Since the land‐use projections do not specify crop types, we use price and yield values of the dominant crop type within each land use as a proxy (2015 data from MapBiomas Project 2023b): soybeans for annual cropland (75%), coffee for perennial (68%), and sugar cane for semi‐perennial (100%). Furthermore, it is assumed that all croplands operate under rainfed conditions, consistent with MapBiomas data from 2015, indicating that approximately 95% of Brazil's agricultural land relies on rainfed conditions (MapBiomas Project 2023b). For pasture, we assume that all areas are dedicated to bovine (ox, cow) carcass production, the major economic output of pastures in Brazil (FAO 2024).

The prices of agricultural product (*P*_*i,t*_) are obtained by adjusting their 2015 prices by indices representing prices changes compared to their 2015 as shown on Equation (3):

(3) $$P_{i,t}=P_{i,2015}\times I_{i,t}$$

where *P*_*i,t*_ is the price of the agricultural products produced by land use *i* at time *t* (USD_2015_/ton), *P*_*i*,2015_ is the price of the agricultural product of land use *i* at 2015 level (USD_2015_/ton), *I*_*i,t*_ is the price index of the agricultural product of land use *i* at time *t* (dimensionless, with *I*_*i*,2015_ equal to 1). *P*_*i*,2015_ is provided by CONAB, the Brazilian National Supply Company, at the state level on a monthly basis in BRL. For this analysis, the annual average prices of soybeans, coffee, sugarcane, and cattle are used, converted to USD using the 2015 FAO exchange rate (FAO 2024). When state‐level data is unavailable, regional averages are assigned to each state. The price indices for each scenario and timestep are sourced from IMAGE (Stehfest et al. 2014).

Regarding yields, different methodologies are applied to croplands and pastures. The yield of cropland *i* at time *t* (ton/ha/year) is determined by Equation (4):

(4) $$Y_{i,t}={\mathrm{PY}}_{i,t}\times M_{i,t}\times {\mathrm{CI}}_{i,t}$$

where *Y*_*i,t*_ is the yield of cropland *i* at time *t* (ton/ha/year), PY_*i,t*_ is the potential yield of crop *i* at time *t* (ton/ha/year), *M*_*i,t*_ is the management factor of cropland *i* at time *t* (dimensionless), and CI_*i,t*_ is the cropping intensity factor for crop *i* at time *t* (dimensionless). Cropping intensity accounts for differences between arable land and harvested area, including factors such as double cropping and fallow land. The management factor reflects differences between potential and actual yields, incorporating variables such as fertilizer and pesticide use, farmer expertise, and data inconsistencies. These data are provided by IMAGE for each scenario and timestep (Stehfest et al. 2014).

For pasture, the yield of beef carcass at time *t* (ton of carcass/ha/year) is determined by Equation (5):

(5) $$Y_{t}=\frac{{\mathrm{PG}}_{t}\times {\mathrm{GI}}_{t}\;}{\;F_{t}}$$

where PG_*t*_ is the potential yield of grass at time *t* (ton dm/ha/year), GI_*t*_ is the grazing intensity at time *t* (dimensionless), and *F*_*t*_ is the feed efficiency factor for intensive systems at time *t* (ton dm/ton of bovine carcass). Once again, these data for each scenario and timestep are provided by IMAGE (Stehfest et al. 2014). In this study, we assume an intensive grazing system in which grass represents only a portion of the total feed consumed by cattle. Accordingly, we assume that producers supply additional feed to the cattle. Revenues generated from extensive pasture systems are not estimated in this analysis.

### 2.6 Trade‐Offs and Synergies Analysis

**FIGURE 2:**
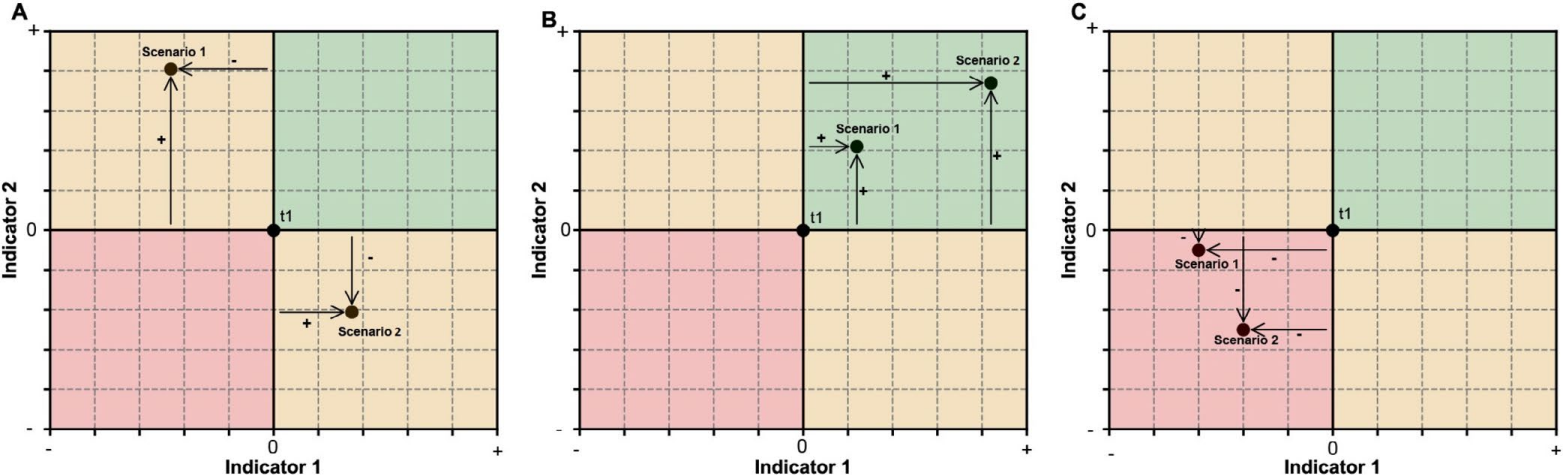
Quantification of trade‐offs and synergies. In (A), examples of trade‐offs are shown. Scenario 1 leads to a decline in indicator 1 while improving indicator 2, compared to the initial state. In contrast, Scenario 2 results in a decline of indicator 2 and an improvement in indicator 1. In (B), synergies are depicted, where both Scenario 1 and Scenario 2 lead to improvements in both indicator 1 and indicator 2 relative to the initial state. Panel (C) illustrates a loss‐loss situation where both indicators decline.

Following the calculation of the indicators, we conduct a trade‐off and synergy analysis for each scenario. At the national level, trade‐offs and synergies are quantified by comparing the evolutions between 2015 and 2050 in: (i) total carbon stock, (ii) the relative change in the size of species distribution areas, calculated as the average across all species compared to their 2015 distributions, and (iii) total agricultural revenue. When two indicators show improvement relative to 2015 levels, this is interpreted as a synergy, indicating progress towards both associated objectives. When one indicator improves while another declines, this reflects a trade‐off, suggesting that gains in one come at the expense of the other. When both indicators decline, this represents a loss–loss outcome. Figure 2 illustrates how these changes in indicators are used to identify and quantify trade‐offs and synergies across the three scenarios.

We map changes in carbon stock, mammal species richness, and agricultural revenue between 2015 and 2050 for each scenario. Each pixel in the resulting maps is then reclassified according to the type of trade‐off or synergy it represents, based on whether each indicator shows a positive, negative, or no change. The most common types of trade‐offs and synergies, in terms of spatial extent, are then mapped, providing insight into the various types of outcomes encountered across Brazil. Additionally, we map the magnitude of these trade‐offs and synergies to highlight where they are most pronounced.

## 3 Results

### 3.1 Trade‐Offs and Synergies

Our results reveal varying trade‐offs and synergies across the different scenarios. SSP1‐1.9 outlines a land‐use trajectory in which natural vegetation expands; whereas SSP2‐4.5 and SSP3‐7.0 follow similar paths characterized by agricultural expansion.

**FIGURE 3:**
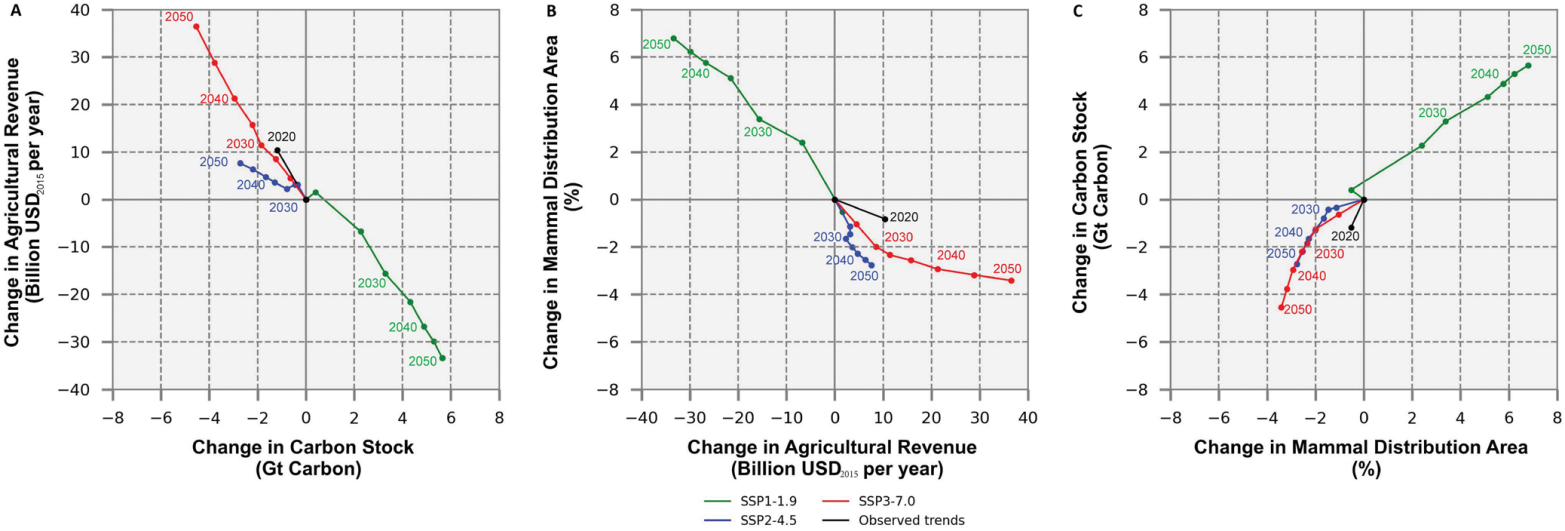
Trade‐offs and synergies at national level. Trade‐offs and synergies between the changes by 2050 in carbon stock (Gt carbon), mammal distribution area (%), and agricultural revenue (USD_2015_/year) compared to their 2015 level for three land‐use change scenarios, as well as for the observed land‐use changes from 2015 to 2020. Panel (A) illustrates a trade‐off between carbon stock and agricultural revenue with improvements in carbon stock under SSP1‐1.9; agricultural revenue increases under SSP2‐4.5 and SSP3‐7.0. Panel (B) shows the trade‐offs between agricultural revenue and mammal distribution areas with an increase in mammal distribution areas under SSP1‐1.9; an increase in agricultural revenue under SSP2‐4.5 and SSP3‐7.0. Panel (C) highlights synergies between mammal distribution area and carbon stock under SSP1‐1.9, while a loss–loss relationship exists under SSP2‐4.5 and SSP3‐7.0.

**FIGURE 4:**
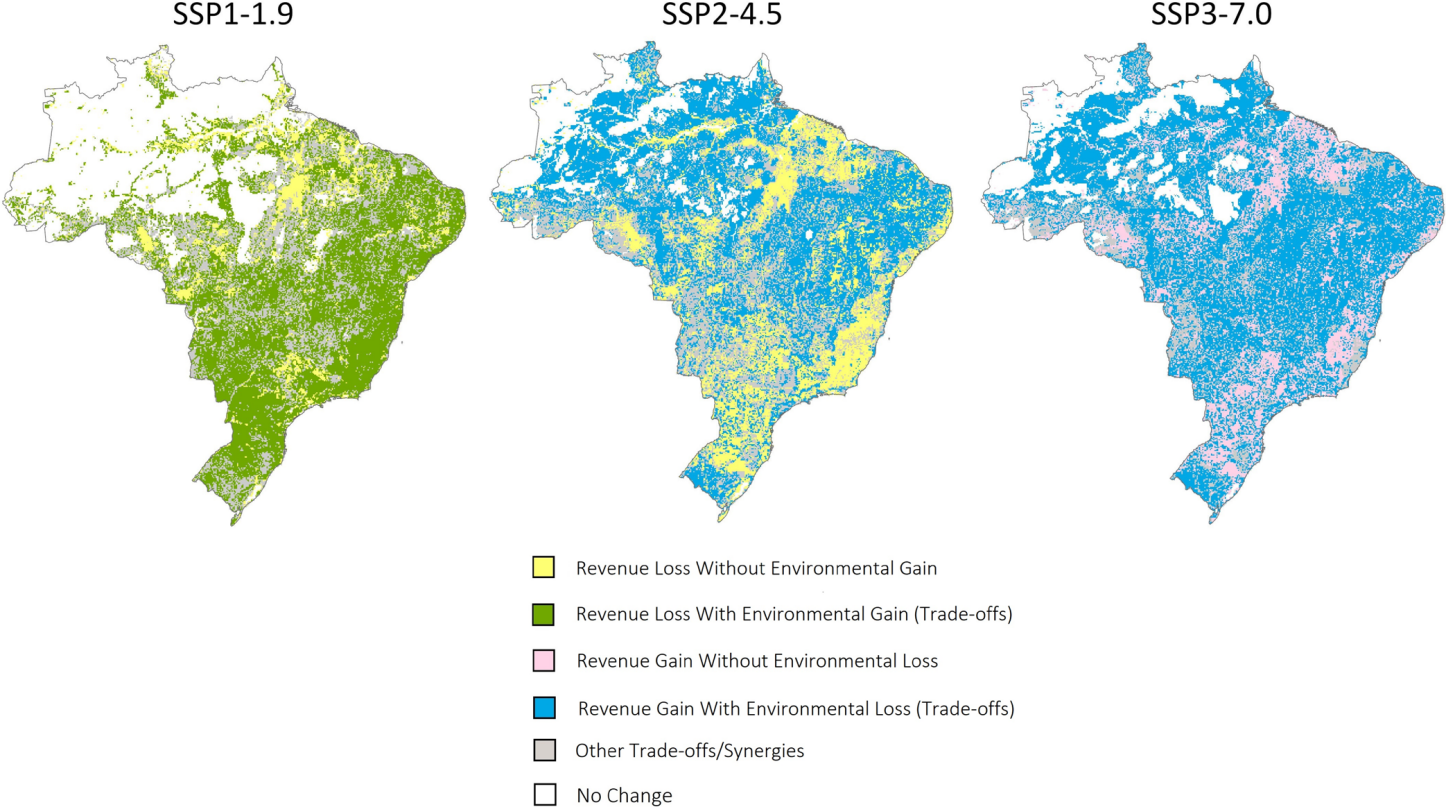
Common types of trade‐offs and synergies across Brazil. These maps display the predominant types of trade‐offs and synergies projected between changes in carbon stock, mammal species richness, and agricultural revenue from 2015 to 2050 across the different scenarios. “Environmental Gain/Loss” refer to change in carbon stock and/or mammal species richness. Map lines delineate study areas and do not necessarily depict accepted national boundaries.

**FIGURE 5:**
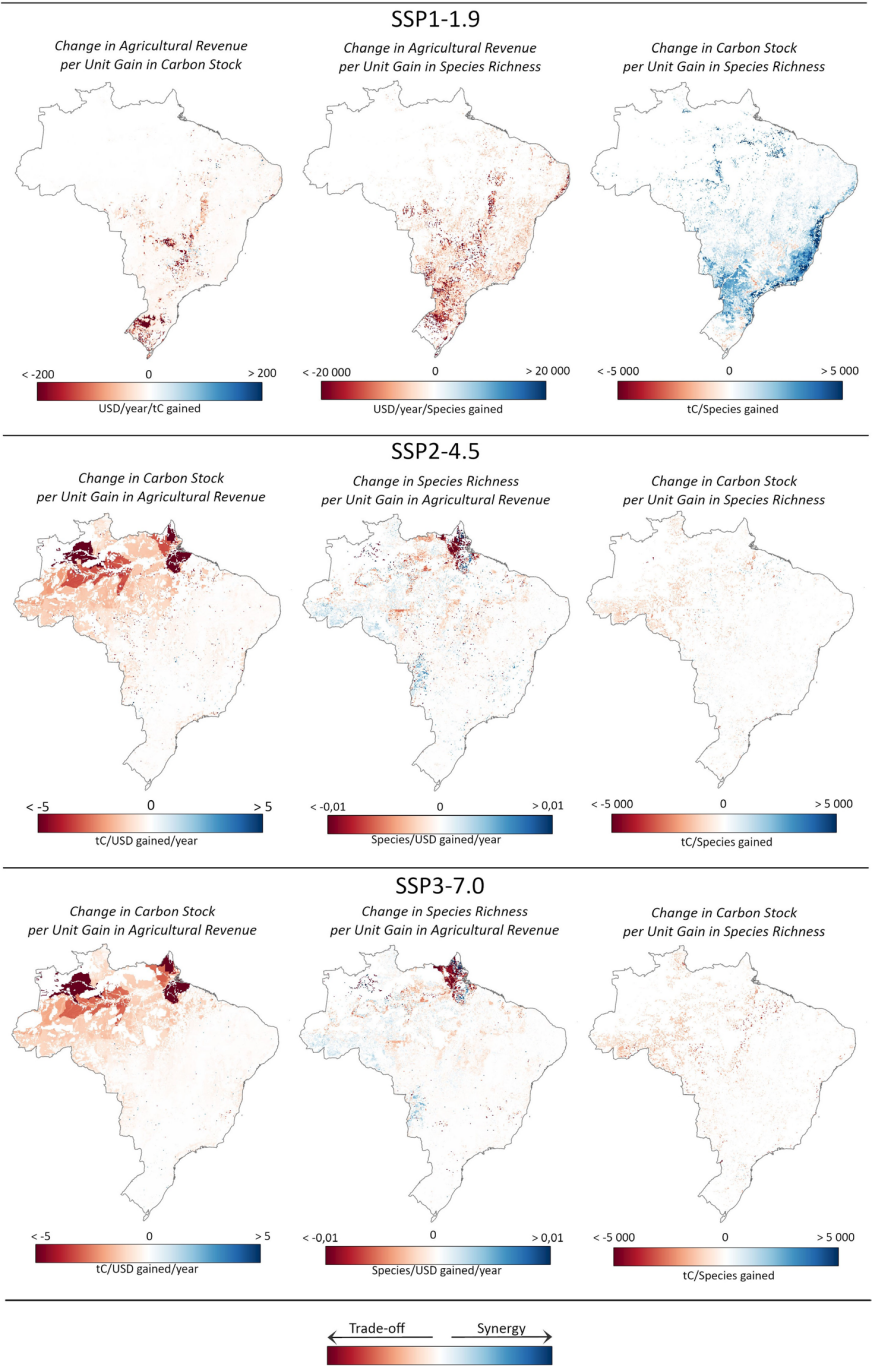
Magnitude of the trade‐offs and synergies across Brazil. These maps display the magnitude of the predominant types of trade‐offs and synergies observed between changes in carbon stock, mammal species richness, and agricultural revenue from 2015 to 2050 across the different scenarios. Map lines delineate study areas and do not necessarily depict accepted national boundaries.

Under the SSP1‐1.9 scenario, both carbon stocks and mammal distribution areas are projected to increase synergistically toward 2050 (Figure 3C, green line). However, this synergy comes with trade‐offs: reduced agricultural revenue (Figure 3A,B, green line). Specifically, each gigaton of land carbon gained between 2015 and 2050 is associated with an annual loss of 5.9 billion USD_2015_ in agricultural revenue. Similarly, each percentage point increase in mammal distribution areas over the same period is projected to result in an annual loss of 4.9 billion USD_2015_ in agricultural revenue. On the other hand, each percentage point increase in mammal distribution area is synergistically associated with a gain of 0.83 gigatons of land carbon. These trade‐offs and synergies are observed throughout Brazil under SSP1‐1.9 (Figure 4), with the most pronounced trade‐offs in areas currently dominated by dense croplands (Figure 5). In these areas, cropland contraction and declining agricultural prices are projected to cause revenue losses while offering only modest gains in carbon storage and species richness. In contrast, reforestation in the Atlantic Forest and in the Amazon rainforest is projected to enhance synergistically both carbon stocks and species richness.

Scenarios SSP2‐4.5 and SSP3‐7.0 project increasing agricultural revenues, reflecting economic growth in the sector (Figure 3A,B, blue and red lines). Yet, these economic gains come with trade‐offs: reduced carbon stocks and a contraction in mammal distribution areas, a loss–loss (Figure 3C, blue and red lines). Specifically, for every billion USD_2015_ of agricultural revenue gained annually between 2015 and 2050, SSP2‐4.5 is projected to result in a loss of 0.36 Gt of carbon, compared to 0.12 Gt under SSP3‐7.0. Likewise, mammal distribution areas are projected to shrink by 0.36% for each billion USD_2015_ gained under SSP2‐4.5 between 2015 and 2050, while the contraction is more modest (0.09%) under SSP3‐7.0. Although land use change under SSP3‐7.0 has a larger overall impact on carbon stocks and mammal distribution areas, the trade‐offs are more severe under SSP2‐4.5. This is primarily due to the decline in agricultural product prices, which limits revenue gains from cropland expansion under SSP2‐4.5 and amplifies the trade‐offs, meaning there is less economic benefit per unit of carbon emitted or mammal habitat lost. In contrast, increasing prices under SSP3‐7.0 increase agricultural revenues, resulting in comparatively smaller trade‐offs. These trade‐offs are observed across Brazil under SSP2‐4.5 and SSP3‐7.0 (Figure 4), with the largest trade‐offs occurring in the Amazon rainforest (Figure 5), where forest is replaced by agricultural land. In the Amazon, deforestation is projected to induce carbon and species loss but, compared to other biomes, provides only modest gains in agricultural revenue.

Additionally, in both SSP1‐1.9 and SSP2‐4.5, some agricultural regions across the country experience revenue declines solely due to decreasing agricultural product prices (Figure 4). In these areas, land use remains unchanged, and there are no impacts on mammal richness or carbon stocks. Conversely, under SSP3‐7.0, some regions benefit from increased agricultural revenues driven entirely by rising prices, with no changes in land use (Figure 4). In these cases, the economic gains occur without compromising mammal richness or carbon sinks, thus avoiding trade‐offs.

### 3.2 Carbon Stock, Species Richness, and Agricultural Revenue Across the Scenarios

The SSP1‐1.9 scenario outlines a land‐use trajectory in which natural vegetation is not only preserved but also expanded, while agricultural land simultaneously declines (Table 1). In this scenario, Brazil's carbon stock is projected to increase by approximately 5.6 Gt between 2015 and 2050, reaching a total of 105 Gt of carbon in 2050. This represents an annual CO_2_ sequestration of around 0.59 Gt, twice as much as Brazil's annual CO_2_ emissions from land use in the early 2010s (MCTI 2020a). Most of this carbon sequestration is projected to occur between 2020 and 2035 (Figure 3), corresponding to a peak in reforestation. Similarly, mammal distribution areas are projected to increase by, on average, 6.8% between 2015 and 2050, also peaking between 2020 and 2035 (Figure 3). However, the SSP1‐1.9 scenario results in agricultural revenue dropping by 33.4 billion USD_2015_/year, representing a reduction of approximately 36% below the 2015 level by 2050. This decline occurs in a context of improved agricultural efficiency and reduced demand for agricultural products (Table 1), which together decrease prices and the need for agricultural land, consequently reducing revenues.

SSP2‐4.5 and SSP3‐7.0 scenarios depict land‐use trajectories distinct from SSP1‐1.9 but similar to each other. They are characterized by agricultural expansion at the expense of natural vegetation (Table 1). These land‐use trajectories result in a decline in Brazil's carbon stock by approximately 2.7 Gt under SSP2‐4.5 and 4.5 Gt under SSP3‐7.0 between 2015 and 2050 (Figure 3). This represents annual emissions of 0.29 Gt under SSP2‐4.5, comparable to Brazil's land‐use CO_2_ emissions in the early 2010s (MCTI 2020a). In contrast, under SSP3‐7.0, emissions are projected to rise to 0.48 Gt per year, exceeding Brazil's early 2010 land‐use CO_2_ emissions (MCTI 2020a). In both scenarios, annual emissions are projected to increase after 2035 due to an intensification of deforestation. Similarly, mammal distribution areas are projected to decrease by 2.7% under SSP2‐4.5 and 3.4% under SSP3‐7.0, on average across species. This contraction in mammal distribution areas is particularly evident between 2015 and 2025. However, these scenarios are beneficial for Brazil's agro‐economic development, with annual revenue rising by 7.6 billion USD_2015_/year under SSP2‐4.5 and 36.5 billion USD_2015_/year under SSP3‐7.0, representing an 8% and 39% increase over 2015 levels by 2050, respectively (Figure 3).

Changes in carbon stock, species richness, and agricultural revenue were also quantified for observed land‐use changes between 2015 and 2020 (MapBiomas Project 2023b). The results indicate a trajectory similar to the SSP2‐4.5 and SSP3‐7.0 scenarios, in which increases in agricultural revenue occur at the expense of carbon stocks and mammal distribution areas. However, agricultural revenue and carbon emissions in 2020 reached levels projected only for 2025 under SSP3‐7.0, driven by a more pronounced agricultural expansion. Despite this accelerated agro‐economic development, the impact on mammal distribution areas in 2020 is comparable to the level projected for that same year under SSP3‐7.0, as agricultural expansion mainly occurred in areas less biodiverse than those affected under SSP3‐7.0.

### 3.3 Spatial Pattern of Carbon Stock, Species Richness, and Agricultural Revenue

**FIGURE 6:**
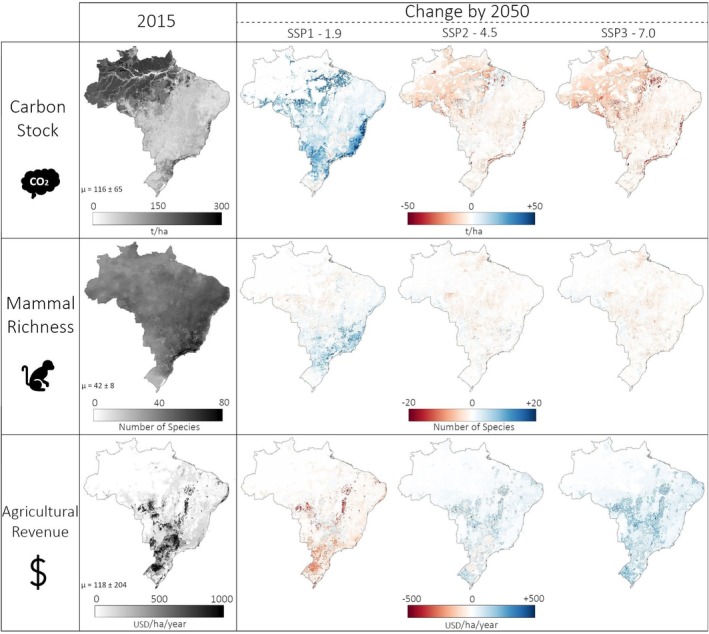
Spatiotemporal impacts of land‐use change. Spatiotemporal changes in carbon stock (t/ha), mammal richness (number of species per cell) and agricultural revenue (USD_2015_/ha/year) between 2015 and 2050 under the three land‐use change scenarios. Map lines delineate study areas and do not necessarily depict accepted national boundaries.

The Amazon rainforest is Brazil's largest carbon hotspot, containing approximately 70% of the country's carbon stock in 2015 (Figure 6). The remaining patches of Atlantic forest along the coast also appear as carbon hotspots, although to a smaller extent. As a result, deforestation in these areas is projected to have a pronounced impact on carbon stocks, as shown under SSP2‐4.5 and SSP3‐7.0 scenarios. Conversely, reforestation in the Amazon and in the Atlantic Forest is projected to result in significant carbon sequestration, as illustrated under SSP1‐1.9. The importance of these two biomes is also illustrated by a peak in carbon sequestration between 2020 and 2035 in SSP1‐1.9 (Figure 3), corresponding to reforestation at the Amazon edge and the Atlantic Forest frontier. Similarly, the intensification of carbon loss after 2035 in SSP2‐4.5 and SSP3‐7.0 (Figure 3) is due to an acceleration in deforestation in the Atlantic Forest. Overall, these results confirm that deforestation of tropical and subtropical forests is a significant source of carbon emissions. Additionally, the regrowth of secondary forests in these areas has a substantial carbon sequestration potential.

The Atlantic forest appears as Brazil's richest biome in terms of mammal species (Figure 6), especially within the remaining forest patches in the southeast of the country. Reforestation, as projected in the SSP1‐1.9 scenario, increases mammal richness in the Atlantic forest and particularly close to its remaining patches of forest, demonstrating the benefit of expanding mammal habitat. On the other hand, the deforestation and agricultural expansion projected in scenarios SSP2‐4.5 and SSP3‐7.0 reduce mammal richness, particularly in the Amazon. Although deforestation is more intense in the SSP3‐7.0 scenario, similar levels of biodiversity are projected under SSP2‐4.5 (Figure 3). This suggests that illegal deforestation in Indigenous Land (ILs), as projected under SSP2‐4.5, particularly contributes to mammal richness loss, while the more intense deforestation is compensated by the protection of ILs under SSP3‐7.0.

In terms of agricultural revenue, regions with dense croplands, particularly in the Cerrado and the South, generate the highest agricultural revenue (Figure 6). While pasture, primarily in the Cerrado and along the Amazon, also contributes to the total agricultural revenue, these areas do not emerge as revenue hotspots due to the larger land area required for meat production. The contraction of agricultural land and the drop in prices under the SSP1‐1.9 scenario are projected to negatively impact agricultural revenue, with the Cerrado experiencing the most significant decline. The South is less affected due to a smaller reduction in agricultural land. Interestingly, under SSP2‐4.5, revenue rises in regions with expanding agricultural land but falls in areas with established agricultural land because the drop in prices is not offset by cropland or pasture expansion in those regions. In contrast, SSP3‐7.0 leads to increased revenue across both new and existing agricultural lands due to a rise in prices (Figure 6).

## 4 Discussion

### 4.1 Balancing Agro‐Economic Development With Biodiversity Preservation and Climate Change Mitigation

Our results show that the magnitude of land‐use change impacts, and consequently, the associated trade‐offs, vary across Brazil and scenarios. This spatially explicit analysis provides valuable insights for decision‐makers by identifying under which conditions and in which areas agricultural expansion is projected to induce the largest trade‐offs. In particular, it highlights that expanding agriculture into forest ecosystems such as the Amazon rainforest and the Atlantic Forest results in the largest conflict between agro‐economic development on the one hand and climate change mitigation and biodiversity preservation on the other.

Both the spatial pattern and the extent of deforestation play important roles in shaping these trade‐offs. While carbon stock loss is primarily driven by the total area deforested, regardless of where it occurs, biodiversity loss is more closely related to the spatial pattern of deforestation. Our results show that deforestation in some biodiverse areas, such as ILs and UCs, particularly reduces the distribution areas of mammal species. This may be due to the presence of species that are sensitive to habitat loss in these areas. In contrast, carbon stock loss is more uniformly associated with the overall scale of deforestation. These findings are consistent with the results of Chaplin‐Kramer et al. (2015), which show that deforestation in the interior of the Amazon is particularly destructive for biodiversity, while its impact on carbon stock is similar to that of the edge of the forest. Therefore, efficient forest protection of ILs and UCs is essential to prevent biodiversity loss, while also contributing to carbon sequestration. Additionally, it highlights opportunities to reduce trade‐offs between agro‐economic development and biodiversity preservation by containing agriculture outside these areas.

The implementation of environmental laws, including the Forest Code, and the protection of UCs and ILs, as illustrated by the SSP1‐1.9 scenario, supports both biodiversity preservation and climate change mitigation. These findings reinforce prior global research indicating that SSP1 represents a distinct pathway that supports both carbon sequestration (e.g., Doelman et al. 2018) and biodiversity preservation (Chaudhary and Mooers 2018; Marquardt et al. 2021). However, enforcing these laws and especially full compliance with mandatory forest restoration on private land is projected to lead to trade‐offs with agro‐economic development, confirming the results of Silva, deCastro Victoria, et al. (2023). Nevertheless, our quantification of these trade‐offs suggests that reforestation around remaining mammal and carbon‐rich areas of the Amazon rainforest and Atlantic Forest is likely to have relatively lower costs on the agricultural economy. For example, in these priority areas, the trade‐offs between agro‐economic development and carbon sequestration are estimated to range between 5 and 10 USD per ton of carbon. By comparison, the carbon price in the European Emissions Trading System is approximately 70 USD per ton (ICAP 2025), indicating opportunities for cost‐effective reforestation. This is particularly relevant given that Brazil has recently launched its own Greenhouse Gas Emissions Trading System (SBCE) (Brasil. 2024). These insights suggest that instead of applying restoration mandates uniformly across all private properties, restoration efforts should strategically target biodiversity‐ and carbon‐rich areas. This is in line with Silva, deCastro Victoria, et al. (2023), who also advocate for a strategic restoration of ecosystems in Brazil, which would benefit biodiversity and carbon sequestration with limited impact on the agricultural economy. Importantly, in some cases, reforestation can be associated with trade‐offs between biodiversity and climate objectives, as shown by Pellegrini et al. (2016). Therefore, restoration efforts should be context‐specific to ensure synergies between these goals.

The broader relevance of our findings is highlighted by global research of, for example, Allan et al. (2022), Dinerstein et al. (2020), Jung et al. (2021) and Strassburg et al. (2014), which underscores the critical role of Brazil in addressing worldwide sustainability challenges. For instance, Jung et al. (2021) identified the Amazon and Atlantic forests as among the most critical regions for global terrestrial biodiversity conservation, carbon storage, and water resource protection, emphasizing their strong potential for synergistic outcomes across multiple environmental global objectives. Strassburg et al. (2020) even identified the border of the Amazon rainforest and Atlantic forest in the top 15% of the regions with the highest global restoration priorities. Yet, these biodiversity‐ and carbon‐rich regions remain under pressure from agricultural expansion. Protecting these areas implies opportunity costs and trade‐offs with agro‐economic development, as the land is also highly suitable for agricultural use. Some research suggests that agricultural intensification and the development of multifunctional landscapes are essential for meeting agricultural demands without harming biodiversity and carbon stocks (e.g., Wolff et al. 2018).

### 4.2 Limitations and Future Research

This research assesses trade‐offs and synergies between agro‐economic development, biodiversity preservation, and climate change mitigation, each represented by a key indicator. However, the contribution of land use to each objective cannot be fully captured by a single indicator. For example, species richness does not reflect species composition, endemism, sensitivity to threat, functional diversity, or rarity, which are also important indicators of biodiversity (Baldwin et al. 2017; Guerin et al. 2015; Guerin and Lowe 2015). In addition, this research focuses just on mammals, while other taxonomic groups might respond differently to land‐use changes (e.g., Pellegrini et al. 2016). Regarding agro‐economic development, revenue provides insight into the agricultural sector's development but neglects production cost, thereby ignoring the profitability of the sector. Including forestry revenue could also reduce the apparent trade‐offs between species richness loss and agricultural revenue gains, particularly in northern Brazil, where forestry is expected to expand under the SSP2‐4.5 and SSP3‐7.0 scenarios. Additionally, this research includes only carbon emissions from land‐use change but not greenhouse gas emissions from agricultural activities such as nitrous oxide or methane emissions from fertilizers and livestock. This results probably in an underestimation of the trade‐off between agro‐economic development and climate change mitigation. Overall, incorporating a broader range of indicators for each objective would allow for a more exhaustive understanding of the impacts and trade‐offs of land use change. Nevertheless, the general pattern of the impact is expected to remain consistent, and these three indicators already offer a clear framework for analyzing trade‐offs and synergies. Future research could also incorporate additional sustainability objectives, such as water availability and quality, to further broaden the scope.

While this research focuses on the impacts of land‐use change, climate change also affects land carbon stock (UNCCD 2017), mammal richness (Carvalho et al. 2019; de Oliveira et al. 2023; Nunez et al. 2019) and agricultural yield (Zilli et al. 2020). Additionally, some studies suggest that deforestation in the Amazon could alter the climate and reduce precipitation not only within the Amazon but also in surrounding areas, thereby impacting agricultural yields and leading to significant agro‐economic losses (Leite‐Filho et al. 2021; O'Connor et al. 2021; Oliveira et al. 2013; Spera et al. 2016). If deforestation in the Amazon continues and pushes the biome to a tipping point, it could transition to a savanna, resulting in drastic agro‐economic, biodiversity, and climate impacts across the entire region (Banerjee et al. 2022; Flores et al. 2024). In this research, we focused on the direct impact of land‐use change to isolate these effects. However, future research could incorporate the effects of climate change and deforestation (Boit et al. 2016; Zhang et al. 2015). Such an approach would provide a more comprehensive understanding of future trade‐offs and synergies and reveal some interactions between land‐use and climate change impacts.

In our results, the Atlantic Forest is identified as Brazil's mammal richest biome, while the literature suggests that the Amazon is another biome rich in mammal species (Lumbierres et al. 2022; Marsh et al. 2022). The underestimation of mammal richness in the Amazon in our study arises from the limited number of species records for that biome in our dataset. Most biodiversity research institutes in Brazil are based in the Atlantic Forest, explaining the lack of records in other biomes (Brito et al. 2009). This results in an underestimation of the trade‐offs between agro‐economic development and biodiversity preservation in this region. To improve the interpretation and spatial comparison of mammal richness, future research should develop methods that are less sensitive to the quantity of records. Additionally, improved species records availability in the Amazon would be highly valuable to support biodiversity preservation in this area.

This research identified and quantified trade‐offs and synergies across three alternative future land use scenarios, supporting informed decisions regarding future land use. These trade‐offs and synergies are tied to the specific scenarios and their respective projected land‐use configurations. Consequently, it remains unclear how far these configurations are from the “best possible” outcomes for each objective. To address this limitation, future research could incorporate a spatial optimization approach, as demonstrated by Verstegen et al. (2017). This approach allows for the optimization of the land‐use configuration with respect to diverse objectives, for example, biodiversity preservation, climate change mitigation, and agro‐economic development. Trade‐offs among diverse objectives can then be assessed by constructing a Pareto front, representing all potential optimal land‐use configurations (Seppelt et al. 2013). Within these optimal configurations, the best solution depends on the relative importance (i.e., weighting) of the objectives. Comparing our findings with a Pareto front could help assess the optimality of the land‐use projections (Seppelt et al. 2013; Verstegen et al. 2017).

## 5 Conclusion

Our research is among the first to quantitatively and spatially assess the trade‐offs and synergies between agro‐economic development, biodiversity preservation, and climate change mitigation at the scale of Brazil, offering insights for future land‐use planning. Our results show that the agricultural economy is projected to grow at the expense of biodiversity preservation and climate change mitigation, and vice versa. These trade‐offs and synergies are caused by changes in natural vegetation and agricultural land, driven by a shifting demand for agricultural products. In particular, under the SSP3‐7.0 scenario, the rising demand for agricultural products between 2015 and 2050 is projected to lead to agricultural expansion into natural land. This pathway is expected to increase Brazil's annual agricultural revenue by $36.5 billion USD_2015_, but results in a 4.5‐Gt reduction in carbon stocks and a 3.4% decline in mammal distribution areas. In contrast, the SSP1‐1.9 scenario projects a reduction in agricultural demand over the same period, resulting in the reconversion of agricultural land to natural vegetation cover. This transition is expected to increase Brazil's carbon stocks by 5.6 Gt and expand mammal distribution areas by 6.8%, though it reduces the annual agricultural revenue by $33.4 billion USD_2015_.

Our findings underscore the potential to reduce trade‐offs by limiting agricultural expansion in biodiversity‐rich and carbon‐rich areas, such as Indigenous Lands (ILs) and Conservation Units (UCs), while also prioritizing the restoration of these regions. These strategies offer a pathway to better harmonize agro‐economic development with biodiversity preservation and climate change mitigation. However, when developing sustainable future land‐use policies, it is essential to consider the global demand for agricultural products and Brazil's role in their supply. The challenge of allocating agricultural land, preventing deforestation, and restoring land will be particularly significant if the demand for agricultural products continues to grow.

## Author Contributions

**Thomas M. R. Gérard:** conceptualization, formal analysis, methodology, software, visualization, writing – original draft. **Sietze J. Norder:** conceptualization, methodology, supervision, writing – review and editing. **Judith A. Verstegen:** conceptualization, methodology, supervision, writing – review and editing. **Jonathan C. Doelman:** methodology, resources, writing – review and editing. **Stefan C. Dekker:** conceptualization, methodology, supervision, writing – review and editing. **Floor van der Hilst:** conceptualization, funding acquisition, methodology, project administration, supervision, writing – review and editing.

## Conflicts of Interest

The authors declare no conflicts of interest.

## Supporting information


**Data S1:** gcb70418‐sup‐0001‐Supinfo.docx.

## Data Availability

The data and scripts supporting the findings of this study are openly available on Zenodo at https://doi.org/10.5281/zenodo.16529938 and https://doi.org/10.5281/zenodo.15497375. Land use projections were obtained from Zenodo at https://zenodo.org/records/5123560. Species occurrence records were obtained from three online databases: GBIF (https://www.gbif.org), SpeciesLink (https://specieslink.net), and SALVE (https://salve.icmbio.gov.br). Topographic data were obtained from the Earth Resources Observation and Science (EROS) Center and are available at https://doi.org/10.5066/F7PR7TFT. Climate data were obtained from WorldClim at https://www.worldclim.org/data/index.html (version 2.1). Annual Mapping of Soil Organic Carbon Stocks, Annual Land Cover and Land Use Maps were obtained from the MapBiomas Project at https://doi.org/10.58053/MapBiomas/DHAYLZ and https://doi.org/10.58053/MAPBIOMAS/VJIJCL, respectively.
