# Supplementary material for: Trade‐Offs and Synergies Between Climate Change Mitigation, Biodiversity Preservation, and Agro‐Economic Development Across Future Land‐Use Scenarios in Brazil
Source: Glob Chang Biol. 2025 Aug 7;31(8):e70418. doi: 10.1111/gcb.70418 (PMC12329712; doi:10.1111/gcb.70418)
Supplement: Supplementary file 1 — Data S1: gcb70418‐sup‐0001‐Supinfo.docx. [file GCB-31-e70418-s001.docx]

Supplementary Material

# Land use scenarios

## Subdivision of Cropland

The "cropland" land use category is subdivided into temporary, perennial, and semi-perennial croplands based on their distribution observed in 2015. To achieve this, we use the 2015 land use map from the MapBiomas project (MapBiomas Project, 2023b). We calculate the proportion of each cropland type within every Brazilian state (Table 1), and these proportions are then used to redistribute the overall cropland fraction into its respective subcategories. It is assumed that these state-level proportions remain constant from 2015 through 2050. The original land use categories from MapBiomas are aggregated as detailed in Table 2.

**Table 1:** Proportion of temporary, perennial and semi-perennial croplands across Brazil’ states in 2015

| **States** | **Temporary Croplands** | **Perennial Croplands** | **Semi perennial Croplands** |
| --- | --- | --- | --- |
| **AC** | 100.0 % | 0.0 % | 0.0 % |
| **AL** | 0.7 % | 0.0 % | 99.3 % |
| **AM** | 100.0 % | 0.0 % | 0.0 % |
| **AP** | 100.0 % | 0.0 % | 0.0 % |
| **BA** | 95.6 % | 4.4 % | 0.0 % |
| **CE** | 48.6 % | 51.4 % | 0.0 % |
| **DF** | 100.0 % | 0.0 % | 0.0 % |
| **ES** | 15.2 % | 84.8 % | 0.0 % |
| **GO** | 85.4 % | 0.0 % | 14.6 % |
| **MA** | 100.0 % | 0.0 % | 0.0 % |
| **MG** | 60.6 % | 21.1 % | 18.3 % |
| **MS** | 82.3 % | 0.0 % | 17.7 % |
| **MT** | 99.1 % | 0.0 % | 0.9 % |
| **PA** | 80.0 % | 20.0 % | 0.0 % |
| **PB** | 7.7 % | 0.0 % | 92.3 % |
| **PE** | 25.6 % | 14.0 % | 60.5 % |
| **PI** | 100.0 % | 0.0 % | 0.0 % |
| **PR** | 90.2 % | 0.6 % | 9.2 % |
| **RJ** | 100.0 % | 0.0 % | 0.0 % |
| **RN** | 87.3 % | 7.0 % | 5.6 % |
| **RO** | 100.0 % | 0.0 % | 0.0 % |
| **RR** | 100.0 % | 0.0 % | 0.0 % |
| **RS** | 100.0 % | 0.0 % | 0.0 % |
| **SC** | 100.0 % | 0.0 % | 0.0 % |
| **SE** | 86.8 % | 0.0 % | 13.2 % |
| **SP** | 19.2 % | 5.3 % | 75.5 % |
| **TO** | 100.0 % | 0.0 % | 0.0 % |

***Table 2 :*** *Matching of land use categories of Mapbiomass* (MapBiomas Project, 2023b) *and land use projection land use* (Silva Bezerra et al., 2022)

| **Land use of the projections** | **Land use categories of Mapbiomas (collection 7.1)** |
| --- | --- |
| **Forest** | Forest Formation |
| **Grassland** | Savana Formation, wetland, Grasslands and other non-forested areas |
| **Planted Pasture** | Pasture |
| **Cropland** | Temporary Cropland (Soybeans, rice, cotton and mosaic of crop)  Perennial Cropland (coffee, citrus and other perennial crop)  Semi-perennial Cropland (Sugarcane) |
| **Forest Plantation** | Forest Plantation |
| **Others** | Beach and Dunes, Urban infrastructure, Rocky outcrop, mining, Aquaculture, river, lake, ocean, salt flat, wooded Sandbanks vegetation, Herbaceous sandbank vegetation, other non-vegetated area, Mangroves |

## Subdivision of Mosaic

The "Mosaic" land use category is disaggregated into specific land use types using a combination of datasets. We use the MapBiomas land use map (MapBiomas Project, 2023b), which provides more detailed land use classifications at a finer spatial resolution than the land use projections. Additionally, we use the 2014 land use map from IBGE (IBGE, 2022), which serves as the basis for the land use projections and includes the Mosaic category. By overlaying the IBGE map with the MapBiomas map, we calculate the proportion of each land use type within areas classified as Mosaic. This analysis is performed separately for each Brazilian mesoregion. The resulting proportions are then used to redistribute the Mosaic category into its respective subcategories. These proportions are assumed to remain constant within each mesoregion from 2015 to 2050. The final subdivisions are presented in Table 3.

**Table 3:** Subdivision of mosaic across Brazil’s mesoregions

| Mesoregion | Forest | Grassland | Pasture | Cropland | Forest Plantation | Other |  | Mesoregion | | | Forest | Grassland | | | Pasture | Cropland | | Forest Plantation | | | Other |
| --- | --- | --- | --- | --- | --- | --- | --- | --- | --- | --- | --- | --- | --- | --- | --- | --- | --- | --- | --- | --- | --- |
| Madeira-Guaporé | 44.01% | 1.26% | 53.66% | 0.03% | 0.00% | 1.04% |  | Oeste de Minas | | | 19.07% | 1.96% | | | 60.85% | 13.46% | | 3.71% | | | 0.95% |
| Leste Rondoniense | 35.69% | 3.33% | 59.30% | 0.98% | 0.01% | 0.69% |  | Sul/Sudoeste de Minas | | | 17.29% | 0.69% | | | 55.27% | 24.87% | | 0.89% | | | 0.99% |
| Vale do Juruá | 64.20% | 0.58% | 32.92% | 0.00% | 0.00% | 2.30% |  | Campo das Vertentes | | | 18.11% | 3.91% | | | 62.84% | 11.65% | | 2.28% | | | 1.20% |
| Vale do Acre | 41.99% | 0.22% | 56.98% | 0.03% | 0.00% | 0.78% |  | Zona da Mata | | | 22.22% | 0.05% | | | 65.19% | 10.68% | | 0.89% | | | 0.96% |
| Norte Amazonense | 88.71% | 1.95% | 1.68% | 0.00% | 0.00% | 7.66% |  | Noroeste Espírito-santense | | | 15.61% | 0.08% | | | 66.98% | 11.87% | | 1.04% | | | 4.42% |
| Sudoeste Amazonense | 70.45% | 2.32% | 18.85% | 0.00% | 0.00% | 8.38% |  | Litoral Norte Espírito-santense | | | 14.77% | 0.97% | | | 61.02% | 15.69% | | 5.59% | | | 1.97% |
| Centro Amazonense | 64.68% | 5.96% | 14.10% | 0.05% | 0.00% | 15.22% |  | Central Espírito-santense | | | 39.34% | 0.40% | | | 39.10% | 18.00% | | 0.90% | | | 2.26% |
| Sul Amazonense | 54.61% | 1.73% | 40.56% | 0.01% | 0.00% | 3.09% |  | Sul Espírito-santense | | | 22.81% | 0.24% | | | 56.31% | 17.93% | | 0.78% | | | 1.94% |
| Norte de Roraima | 50.45% | 10.62% | 38.04% | 0.24% | 0.00% | 0.66% |  | Noroeste Fluminense | | | 11.57% | 0.00% | | | 83.54% | 3.86% | | 0.07% | | | 0.94% |
| Sul de Roraima | 53.90% | 2.71% | 41.77% | 0.07% | 0.00% | 1.54% |  | Norte Fluminense | | | 20.43% | 1.46% | | | 66.67% | 9.32% | | 0.01% | | | 2.11% |
| Baixo Amazonas | 66.89% | 2.78% | 23.32% | 2.33% | 0.67% | 4.00% |  | Centro Fluminense | | | 31.34% | 0.01% | | | 57.88% | 8.31% | | 0.36% | | | 2.10% |
| Marajó | 59.68% | 6.45% | 4.91% | 0.00% | 0.00% | 28.96% |  | Baixadas | | | 15.97% | 1.23% | | | 69.16% | 9.52% | | 0.00% | | | 4.11% |
| Metropolitana de Belém | 57.06% | 0.73% | 27.24% | 0.02% | 0.02% | 14.94% |  | Sul Fluminense | | | 28.28% | 0.04% | | | 61.38% | 7.49% | | 0.14% | | | 2.66% |
| Nordeste Paraense | 62.16% | 1.94% | 24.64% | 0.02% | 0.06% | 11.17% |  | Metropolitana do Rio de Janeiro | | | 27.27% | 1.24% | | | 50.91% | 13.90% | | 0.03% | | | 6.64% |
| Sudoeste Paraense | 67.46% | 0.90% | 30.17% | 0.09% | 0.00% | 1.38% |  | São José do Rio Preto | | | 9.28% | 0.92% | | | 39.33% | 49.43% | | 0.18% | | | 0.86% |
| Sudeste Paraense | 47.13% | 1.81% | 49.63% | 0.28% | 0.13% | 1.02% |  | Ribeirão Preto | | | 14.26% | 2.65% | | | 24.91% | 56.55% | | 0.57% | | | 1.07% |
| Norte do Amapá | 38.18% | 51.84% | 2.02% | 0.00% | 0.01% | 7.95% |  | Araçatuba | | | 10.24% | 1.44% | | | 43.98% | 43.27% | | 0.13% | | | 0.94% |
| Sul do Amapá | 61.26% | 19.55% | 8.06% | 0.04% | 4.00% | 7.10% |  | Bauru | | | 13.20% | 2.90% | | | 38.56% | 39.77% | | 4.48% | | | 1.08% |
| Ocidental do Tocantins | 34.34% | 23.99% | 38.56% | 1.56% | 0.20% | 1.35% |  | Araraquara | | | 14.69% | 3.65% | | | 20.58% | 59.43% | | 0.78% | | | 0.88% |
| Oriental do Tocantins | 17.72% | 54.56% | 20.38% | 5.36% | 0.07% | 1.91% |  | Piracicaba | | | 16.18% | 2.53% | | | 33.33% | 42.48% | | 4.24% | | | 1.25% |
| Norte Maranhense | 57.89% | 11.92% | 14.55% | 1.25% | 0.00% | 14.38% |  | Campinas | | | 16.97% | 0.16% | | | 50.05% | 28.35% | | 2.39% | | | 2.08% |
| Oeste Maranhense | 45.62% | 2.57% | 47.43% | 0.58% | 0.75% | 3.05% |  | Presidente Prudente | | | 13.51% | 1.90% | | | 61.80% | 21.90% | | 0.29% | | | 0.61% |
| Centro Maranhense | 46.03% | 6.79% | 39.69% | 6.12% | 0.10% | 1.26% |  | Marília | | | 16.61% | 1.38% | | | 56.54% | 23.75% | | 1.13% | | | 0.59% |
| Leste Maranhense | 67.76% | 17.15% | 10.00% | 4.37% | 0.36% | 0.36% |  | Assis | | | 13.84% | 2.10% | | | 38.53% | 41.58% | | 2.72% | | | 1.22% |
| Sul Maranhense | 17.16% | 65.05% | 12.89% | 4.02% | 0.07% | 0.81% |  | Itapetininga | | | 25.93% | 1.95% | | | 40.10% | 22.90% | | 8.23% | | | 0.90% |
| Norte Piauiense | 50.14% | 38.89% | 8.97% | 1.18% | 0.01% | 0.80% |  | Macro Metropolitana Paulista | | | 29.45% | 0.11% | | | 35.05% | 25.80% | | 4.76% | | | 4.82% |
| Centro-Norte Piauiense | 20.82% | 51.22% | 21.87% | 3.88% | 0.02% | 2.19% |  | Vale do Paraíba Paulista | | | 22.19% | 0.00% | | | 60.91% | 12.11% | | 1.89% | | | 2.90% |
| Sudoeste Piauiense | 18.24% | 63.33% | 12.40% | 5.35% | 0.01% | 0.67% |  | Litoral Sul Paulista | | | 59.52% | 0.90% | | | 15.39% | 13.84% | | 0.16% | | | 10.19% |
| Sudeste Piauiense | 0.49% | 54.85% | 34.38% | 8.74% | 0.00% | 1.54% |  | Metropolitana de São Paulo | | | 42.37% | 0.29% | | | 25.24% | 20.36% | | 2.31% | | | 9.44% |
| Noroeste Cearense | 31.23% | 31.83% | 16.69% | 18.30% | 0.00% | 1.94% |  | Noroeste Paranaense | | | 9.12% | 1.13% | | | 60.48% | 28.38% | | 0.22% | | | 0.67% |
| Norte Cearense | 28.94% | 35.79% | 14.38% | 18.69% | 0.00% | 2.20% |  | Centro Ocidental Paranaense | | | 19.10% | 0.00% | | | 34.68% | 44.39% | | 1.13% | | | 0.70% |
| Metropolitana de Fortaleza | 47.03% | 19.55% | 11.87% | 15.58% | 0.00% | 5.97% |  | Norte Central Paranaense | | | 19.23% | 0.04% | | | 38.16% | 40.36% | | 1.30% | | | 0.91% |
| Sertões Cearenses | 1.12% | 58.42% | 35.23% | 4.80% | 0.00% | 0.44% |  | Norte Pioneiro Paranaense | | | 16.99% | 0.09% | | | 52.98% | 26.59% | | 2.58% | | | 0.77% |
| Jaguaribe | 3.99% | 31.39% | 44.10% | 18.54% | 0.00% | 1.98% |  | Centro Oriental Paranaense | | | 38.21% | 1.45% | | | 25.92% | 24.03% | | 9.38% | | | 1.01% |
| Centro-Sul Cearense | 2.38% | 50.75% | 44.14% | 1.64% | 0.00% | 1.08% |  | Oeste Paranaense | | | 23.79% | 0.08% | | | 33.15% | 40.96% | | 1.10% | | | 0.93% |
| Sul Cearense | 23.82% | 43.52% | 28.93% | 3.04% | 0.00% | 0.69% |  | Sudoeste Paranaense | | | 24.31% | 0.00% | | | 34.71% | 38.21% | | 1.81% | | | 0.95% |
| Oeste Potiguar | 1.47% | 46.79% | 37.61% | 11.97% | 0.00% | 2.16% |  | Centro-Sul Paranaense | | | 40.29% | 0.29% | | | 29.48% | 25.27% | | 3.87% | | | 0.79% |
| Central Potiguar | 0.22% | 43.51% | 42.58% | 11.69% | 0.00% | 2.00% |  | Sudeste Paranaense | | | 51.28% | 0.67% | | | 13.27% | 26.82% | | 7.17% | | | 0.79% |
| Agreste Potiguar | 0.07% | 38.87% | 49.69% | 10.52% | 0.00% | 0.85% |  | Metropolitana de Curitiba | | | 51.58% | 0.79% | | | 16.89% | 18.53% | | 6.98% | | | 5.23% |
| Leste Potiguar | 3.59% | 16.44% | 40.28% | 33.98% | 0.00% | 5.70% |  | Oeste Catarinense | | | 34.23% | 0.05% | | | 26.98% | 31.06% | | 6.50% | | | 1.17% |
| Sertão Paraibano | 0.00% | 60.74% | 37.13% | 1.27% | 0.00% | 0.86% |  | Norte Catarinense | | | 51.57% | 0.36% | | | 13.78% | 20.04% | | 8.14% | | | 6.12% |
| Borborema | 0.01% | 39.53% | 54.73% | 4.33% | 0.00% | 1.40% |  | Serrana | | | 46.88% | 9.03% | | | 20.71% | 13.69% | | 8.60% | | | 1.09% |
| Agreste Paraibano | 0.30% | 33.71% | 61.69% | 3.18% | 0.00% | 1.11% |  | Vale do Itajaí | | | 56.32% | 0.00% | | | 18.33% | 16.38% | | 6.82% | | | 2.14% |
| Mata Paraibana | 11.52% | 6.15% | 39.56% | 40.62% | 0.02% | 2.12% |  | Grande Florianópolis | | | 63.87% | 0.57% | | | 19.07% | 8.15% | | 6.48% | | | 1.87% |
| Sertão Pernambucano | 3.14% | 50.11% | 41.85% | 4.27% | 0.00% | 0.63% |  | Sul Catarinense | | | 40.25% | 0.80% | | | 33.09% | 17.34% | | 3.51% | | | 5.01% |
| São Francisco Pernambucano | 0.34% | 48.77% | 36.22% | 10.87% | 0.00% | 3.80% |  | Noroeste Rio-grandense | | | 28.97% | 4.89% | | | 17.07% | 46.34% | | 1.58% | | | 1.15% |
| Agreste Pernambucano | 3.14% | 20.59% | 70.55% | 4.48% | 0.00% | 1.24% |  | Nordeste Rio-grandense | | | 53.02% | 8.06% | | | 13.24% | 19.01% | | 5.48% | | | 1.19% |
| Mata Pernambucana | 13.49% | 1.05% | 48.89% | 35.16% | 0.00% | 1.41% |  | Centro Ocidental Rio-grandense | | | 39.73% | 25.50% | | | 7.55% | 24.02% | | 1.46% | | | 1.74% |
| Metropolitana de Recife | 27.59% | 2.52% | 24.68% | 40.66% | 0.02% | 4.55% |  | Centro Oriental Rio-grandense | | | 42.84% | 4.18% | | | 15.61% | 26.65% | | 9.38% | | | 1.33% |
| Sertão Alagoano | 0.32% | 28.42% | 69.28% | 1.39% | 0.00% | 0.59% |  | Metropolitana de Porto Alegre | | | 44.19% | 27.60% | | | 7.04% | 10.02% | | 7.69% | | | 3.45% |
| Agreste Alagoano | 2.62% | 17.20% | 72.63% | 6.44% | 0.00% | 1.10% |  | Sudoeste Rio-grandense | | | 15.79% | 42.62% | | | 3.95% | 33.10% | | 0.43% | | | 4.11% |
| Leste Alagoano | 14.77% | 1.44% | 56.21% | 25.29% | 0.00% | 2.29% |  | Sudeste Rio-grandense | | | 31.14% | 39.28% | | | 5.01% | 16.39% | | 5.66% | | | 2.52% |
| Sertão Sergipano | 0.06% | 23.08% | 72.25% | 3.73% | 0.00% | 0.88% |  | Lagoa Mirim | | | 0.00% | 64.47% | | | 3.79% | 30.36% | | 0.02% | | | 1.36% |
| Agreste Sergipano | 2.55% | 7.52% | 80.17% | 9.29% | 0.01% | 0.46% |  | Lagoa dos Patos | | | 11.24% | 36.75% | | | 2.50% | 13.80% | | 4.30% | | | 31.41% |
| Leste Sergipano | 11.83% | 6.63% | 68.59% | 8.76% | 0.40% | 3.80% |  | Pantanais Sul Mato-grossense | | | 30.46% | 55.52% | | | 7.28% | 0.98% | | 0.03% | | | 5.74% |
| Extremo Oeste Baiano | 5.58% | 55.92% | 34.59% | 3.21% | 0.00% | 0.70% |  | Centro Norte de MG do Sul | | | 24.54% | 20.28% | | | 46.59% | 7.69% | | 0.27% | | | 0.64% |
| Vale São-Franciscano da Bahia | 1.49% | 56.45% | 31.12% | 8.93% | 0.00% | 2.00% |  | Leste de Mato Grosso do Sul | | | 20.36% | 21.45% | | | 46.17% | 10.41% | | 1.24% | | | 0.37% |
| Centro Norte Baiano | 7.27% | 38.22% | 41.20% | 12.01% | 0.13% | 1.17% |  | Sudoeste de Mato Grosso do Sul | | | 32.86% | 9.87% | | | 38.36% | 18.10% | | 0.23% | | | 0.58% |
| Nordeste Baiano | 7.03% | 21.09% | 63.38% | 4.99% | 2.52% | 0.99% |  | Norte Mato-grossense | | | 49.14% | 4.54% | | | 35.92% | 9.54% | | 0.15% | | | 0.71% |
| Metropolitana de Salvador | 20.25% | 2.50% | 58.77% | 14.41% | 1.61% | 2.46% |  | Nordeste Mato-grossense | | | 40.90% | 20.57% | | | 32.62% | 5.32% | | 0.04% | | | 0.55% |
| Centro Sul Baiano | 10.18% | 30.04% | 55.53% | 3.77% | 0.10% | 0.38% |  | Sudoeste Mato-grossense | | | 26.81% | 24.17% | | | 42.90% | 2.65% | | 0.17% | | | 3.30% |
| Sul Baiano | 32.27% | 1.48% | 51.77% | 8.46% | 5.13% | 0.89% |  | Centro-Sul Mato-grossense | | | 31.51% | 53.36% | | | 9.66% | 0.68% | | 0.12% | | | 4.68% |
| Noroeste de Minas | 11.05% | 34.32% | 44.51% | 8.02% | 1.00% | 1.10% |  | Sudeste Mato-grossense | | | 19.21% | 28.66% | | | 34.55% | 16.24% | | 0.22% | | | 1.12% |
| Norte de Minas | 3.84% | 42.85% | 47.16% | 4.59% | 0.85% | 0.71% |  | Noroeste Goiano | | | 40.57% | 23.14% | | | 31.77% | 2.76% | | 0.04% | | | 1.72% |
| Jequitinhonha | 35.73% | 13.13% | 40.74% | 7.93% | 1.30% | 1.17% |  | Norte Goiano | | | 15.49% | 42.29% | | | 34.67% | 5.75% | | 0.00% | | | 1.80% |
| Vale do Mucuri | 29.80% | 0.05% | 60.75% | 7.58% | 0.61% | 1.20% |  | Centro Goiano | | | 20.90% | 6.70% | | | 52.30% | 14.31% | | 0.84% | | | 4.95% |
| Triângulo Mineiro | 11.39% | 6.62% | 51.63% | 29.19% | 0.42% | 0.75% |  | Leste Goiano | | | 5.82% | 23.76% | | | 52.99% | 15.27% | | 0.46% | | | 1.70% |
| Central Mineira | 9.07% | 9.52% | 65.50% | 13.00% | 2.14% | 0.77% |  | Sul Goiano | | | 12.17% | 9.01% | | | 43.77% | 33.55% | | 0.41% | | | 1.08% |
| Metropolitana de Belo Horizonte | 37.60% | 4.49% | 45.41% | 8.07% | 1.86% | 2.58% |  | Distrito Federal | | | 14.86% | 21.99% | | | 20.84% | 22.45% | | 0.00% | | | 19.86% |
| Vale do Rio Doce | 25.96% | 1.30% | 62.87% | 7.43% | 1.08% | 1.36% |  |  |  |  | | |  |  | | |  | |  |  |  |

# Carbon stock

## Biomass

The Brazilian Ministry of Innovation and Sciences (MCTI) provided Brazilian-specific AGB and BGB reference values (MCTI, 2020; SIRENE et al., 2020). These values result from an extensive literature review conduct conducted during the fourth Brazilian inventory of anthropogenic emissions and GHG removals. The dataset is available in a spatially explicit format through the SIRENE platform (MCTI, 2020; SIRENE et al., 2020). These reference values are specific to biomes and climates and phytophysiome (i.e. natural vegetation cover without any human interaction). Therefore, these reference values are used for forest and grassland land use, assuming mature systems. For other land uses such as pasture, agriculture, and forest plantations, we use IPCC reference values specific to land use and climate conditions (IPCC, 2006, 2019). Regarding forest plantation, they were assumed to contain 80% of eucalyptus and 20% of pine, based on the 2023 annual report of the Brazilian Tree Industry (Ibá, 2023). For urban areas and other land uses, we assume no carbon biomass (IPCC, 2006, 2019). A summary of the source of the reference values is provided in the table 4 and 5.

**Table 4:** AGB reference values (t/ha)

|  | **Climate zone** | | | | |
| --- | --- | --- | --- | --- | --- |
|  | Warm temperate moist  t/ha | Tropical dry  t/ha | Tropical moist  t/ha | Tropical wet  t/ha | Tropical montane  t/ha |
| **Forest** | MCTI | MCTI | MCTI | MCTI | MCTI |
| **Grassland** | MCTI | MCTI | MCTI | MCTI | MCTI |
| **Pasture** | 1.27 | 1.08 | 2.91 | 2.91 | 1.08 |
| **Forest Plantation** | 77.19 | 27.90 | 58.59 | 102.30 | 60.45 |
| **Temporary Croplands** | 4.70 | 4.70 | 4.70 | 4.70 | 4.70 |
| **Perennial Croplands** | 2.10 | 1.80 | 2.60 | 10.00 | 4.13 |
| **Semi-Perennial Croplands** | 7.84 | 7.84 | 7.84 | 7.84 | 7.84 |
| **Other** | 0.00 | 0.00 | 0.00 | 0.00 | 0.00 |

**Table 5:** BGB reference values (t/ha)

|  | **Climate zone** | | | | |
| --- | --- | --- | --- | --- | --- |
|  | Warm temperate moist  t/ha | Tropical dry  t/ha | Tropical moist  t/ha | Tropical wet  t/ha | Tropical montane  t/ha |
| **Forest** | MCTI | MCTI | MCTI | MCTI | MCTI |
| **Grassland** | MCTI | MCTI | MCTI | MCTI | MCTI |
| **Pasture** | 5.08 | 3.03 | 4.66 | 4.66 | 1.73 |
| **Forest Plantation** | 21.92 | 10.57 | 16.70 | 17.39 | 12.39 |
| **Temporary Croplands** | 0.00 | 0.00 | 0.00 | 0.00 | 0.00 |
| **Perennial Croplands** | 0.00 | 0.00 | 0.00 | 0.00 | 0.00 |
| **Semi-Perennial Croplands** | 1.57 | 1.57 | 1.57 | 1.57 | 1.57 |
| **Other** | 0.00 | 0.00 | 0.00 | 0.00 | 0.00 |

## Soil Organic Carbon (SOC)

This research employs specific SOC reference values (SOC_ref_) for Brazil deriving from the 2015 Mapbiomass solo beta collection, hereafter referred to as the SOC map (MapBiomas Project, 2023a). This collection includes annual maps of the SOC stock in the topsoil (up to a depth of 30 centimeters, aligned with the IPCC guidelines) from 1985 to 2021, with a resolution of 30 meters. The SOC map was overlaid with land use, climate and soil maps to calculate the average SOC per land use, climate and soil type. Those average values were then used as SOC_ref_. The land use and climate zone employed were consistent with those used in producing the SOC map, namely the 2015 Mapbiomas Land Use Map of Brazil Collection 7.1 (MapBiomas Project, 2023b) and Koppen’s climate classification map for Brazil (Alvares et al., 2013), respectively. Regarding the soil map, since no specific soil type map was used in producing the SOC map (MapBiomas Project, 2023a), the IBGE subordem SiBCS soil map was selected to delineate soil type zones (IBGE, 2024)

This research employed the F values_,_ (Tables 7 to 9) of the MCTI (MCTI, 2020; SIRENE et al., 2020). The MCTI land use classification was matched to the one of the projections according to Table 6.

**Table 6:** Matching of IGBE and MCTI land use classification

| **Land use projection categories** | **MCTI classification** |
| --- | --- |
| **Forest vegetation** | Forest (Managed, unmanaged, secondary, selective logging) |
| **Grassland vegetation** | Grassland (Managed, unmanaged, secondary) |
| **Planted Pasture** | Pasture (Natural, planted) |
| **Agriculture** | Annal cropland, Perennial cropland, Semiperennial croplands |
| **Forestry** | Planted forest |
| **Others** | Beach and Dunes, settlement, Rocky outcrop, mining, river, lake, ocean |

**Table 7:** Fc values (dimensionless) (MCTI, 2020; SIRENE et al., 2020)

|  | **Biome** | | | | | |
| --- | --- | --- | --- | --- | --- | --- |
| **Land use** | **Amazonia** | **Cerrado** | **Caatinga** | **Mata Atlantica** | **Pantanal** | **Pampa** |
| **Forest vegetation** | 1.00 | 1.00 | 1.00 | 1.00 | 1.00 | 1.00 |
| **Forestry** | 0.94 | 0.94 | 0.94 | 0.94 | 0.94 | 0.94 |
| **Grassland vegetation** | 1.00 | 1.00 | 1.00 | 1.00 | 1.00 | 1.00 |
| **Pasture** | Table 8 | | | | | |
| **Annual cropland** | Table 9 | | | | | |
| **Perennial croplands** | 0.98 | 0.98 | 0.71 | 0.98 | 0.98 | 0.98 |
| **Semi-perennial croplands** | 0.92 | 092 | 0.92 | 0.92 | 0.92 | 0.92 |
| **Other** | 0.00 | 0.00 | 0.00 | 0.00 | 0.00 | 0.00 |

**Table 8**: Pasture Fc value (dimensionless) (MCTI, 2020; SIRENE et al., 2020). Empty values indicate that the respective state does not occur within that biome.

|  | **Biome** | | | | | |
| --- | --- | --- | --- | --- | --- | --- |
| **States** | **Amazonia** | **Cerrado** | **Caatinga** | **Mata Atlantica** | **Pantanal** | **Pampa** |
| **Acre** | 0.98 |  |  |  |  |  |
| **Alagoas** |  |  | 1.03 | 0.99 |  |  |
| **Amazonas** | 0.99 |  |  |  |  |  |
| **Amapá** | 0.93 |  |  |  |  |  |
| **Bahia** |  | 0.96 | 1.04 | 0.96 |  |  |
| **Ceará** |  |  | 1.00 |  |  |  |
| **Distrito Federal** |  | 0.99 |  |  |  |  |
| **Espírito Santo** |  |  |  | 0.98 |  |  |
| **Goiàs** |  | 0.99 |  | 0.99 |  |  |
| **Maranhão** | 0.98 | 0.98 | 1.07 |  |  |  |
| **Minas Gerais** |  | 0.98 | 1.06 | 0.98 |  |  |
| **Mata Grosso do Sul** |  | 0.99 |  |  | 0.99 |  |
| **Mato Grosso** | 0.98 | 0.98 |  |  | 0.98 |  |
| **Pará** | 0.98 | 0.98 |  |  |  |  |
| **Paraíba** |  |  | 1.01 | 0.97 |  |  |
| **Pernambuco** |  |  | 1.01 | 0.96 |  |  |
| **Piauí** |  | 0.96 | 1.02 |  |  |  |
| **Paraná** |  | 1.00 |  | 1.00 |  |  |
| **Rio de Janeiro** |  |  |  | 0.99 |  |  |
| **Rio Grande do Norte** |  |  | 1.00 | 0.98 |  |  |
| **Rondonia** | 1.00 | 1.00 |  |  |  |  |
| **Roraima** | 0.99 |  |  |  |  |  |
| **Rio Grande do Sul** |  |  |  | 1.00 |  | 1.00 |
| **Santa Catarina** |  |  |  | 0.99 |  |  |
| **Sergipe** |  |  | 1.05 | 0.98 |  |  |
| **São Paulo** |  | 1.00 |  | 1.00 |  |  |
| **Tocantin** | 0.98 | 0.98 |  |  |  |  |

**Table 9:** Temporary crops Fc value (dimensionless) (MCTI, 2020; SIRENE et al., 2020). Empty values indicate that the respective state does not occur within that biome.

|  | **Biome** | | | | | |
| --- | --- | --- | --- | --- | --- | --- |
| **States** | **Amazonia** | **Cerrado** | **Caatinga** | **Mata Atlantica** | **Pantanal** | **Pampa** |
| **Acre** | 0.87 |  |  |  |  |  |
| **Alagoas** |  |  | 0.83 | 0.86 |  |  |
| **Amazonas** | 0.87 |  |  |  |  |  |
| **Amapá** | 0.87 |  |  |  |  |  |
| **Bahia** |  | 0.91 | 0.88 | 0.90 |  |  |
| **Ceará** |  |  | 0.83 |  |  |  |
| **Distrito Federal** |  | 0.91 |  |  |  |  |
| **Espírito Santo** |  |  |  | 0.86 |  |  |
| **Goiàs** |  | 0.91 |  | 0.91 |  |  |
| **Maranhão** | 0.92 | 0.91 | 0.91 |  |  |  |
| **Minas Gerais** |  | 0.91 | 0.88 | 0.90 |  |  |
| **Mata Grosso do Sul** |  | 0.91 |  | 0.90 | 0.90 |  |
| **Mato Grosso** | 0.91 | 0.91 |  |  | 0.91 |  |
| **Pará** | 0.88 | 0.90 |  |  |  |  |
| **Paraíba** |  |  | 0.83 | 0.86 |  |  |
| **Pernambuco** |  |  | 0.83 | 0.86 |  |  |
| **Piauí** |  | 0.91 | 0.88 |  |  |  |
| **Paraná** |  | 0.91 |  | 0.91 |  |  |
| **Rio de Janeiro** |  |  |  | 0.87 |  |  |
| **Rio Grande do Norte** |  |  | 0.83 | 0.86 |  |  |
| **Rondonia** | 0.89 | 0.91 |  |  |  |  |
| **Roraima** | 0.91 |  |  |  |  |  |
| **Rio Grande do Sul** |  |  |  | 0.93 |  | 0.93 |
| **Santa Catarina** |  |  |  | 0.92 |  |  |
| **Sergipe** |  |  | 0.83 | 0.86 |  |  |
| **São Paulo** |  | 0.90 |  | 0.87 |  |  |
| **Tocantin** | 0.90 | 0.91 |  |  |  |  |

# Mammal Richness

## Data Preparation

Occurrence records were extracted, in June 2024, from the online databases: GBIF (*GBIF*, 2024), SpeciesLink (CRIA, 2024) and, SALVE (ICMBio, 2024). Only records of species observed between 1990 and 2022 were kept to ensure they coincide temporally with the environmental variables. For the remaining records, *“CoordinateCleaner”,* an R code tool for cleaning occurrence records from biological databases, was used to eliminate records with incorrect geo-referencing (Zizka et al., 2019). This includes records geo-referenced in the ocean, within urban areas, outside Brazil or in biodiversity institutions. Records with invalid, unrecognized or unprecise coordinates were also removed. Besides, the inconsistency in standardization across online databases regarding taxonomic names could result in records of the same species being named differently. To tackle this issue, the *“rotl”* R package was employed to align records with their taxonomic names as described in the Open Tree of Life (OTL), a digital phylogenetic tree including all organisms and their taxonomic details (Michonneau et al., 2016). Records that could not be matched with an OTL taxonomic name were excluded.

Geographic sampling bias of occurrence records is a common challenge in species distribution studies. If left untreated, it may lead to the over-representation of the environmental condition of the regions oversampled, ultimately undermining model interpretability (Aiello‐Lammens et al., 2015; Fletcher & Fortin, 2018). To improve the model’s performance, spatial thinning was applied to the occurrence records using the *“spThin”* R package (Aiello‐Lammens et al., 2015). This tool randomly selects one record within a certain distance radius. In this research we use a 10km radius (which corresponds to the resolution of the land use projections and environmental variables). Consequently, only one occurrence record was kept per cell of environmental variable. After the thinning process, the Nearest Neighbor Index (NNI) was calculated for each species (Aiello‐Lammens et al., 2015) . Species with a NNI inferior to 0,5, indicating significant Geographic sampling bias, were excluded from the research.

After addressing temporal, coordinate and taxonomic cleaning, as well as mitigating sampling bias, 234 distinct species of mammals remained with an average of 66 records per species (15342 records in total) (figure 1).

Logistic regression models also require absence records, which can be difficult to obtain for species (Barbet‐Massin et al., 2012). Therefore, it is common to use “pseudo-absence data” resulting from a random sampling in areas with habitat unsuitable for the species (Barbet‐Massin et al., 2012; Fletcher & Fortin, 2018). This method has been shown to be the most reliable for the logistic model (Barbet‐Massin et al., 2012; Fletcher & Fortin, 2018), especially when using a large number of pseudo-absence data with equal weighting for presence and absence (Barbet‐Massin et al., 2012). Those recommendations were applied in this research and 1000 pseudo-absence data were randomly sampled in unsuitable areas. Expert species range maps were used to identify unsuitable areas (Marsh et al., 2022).


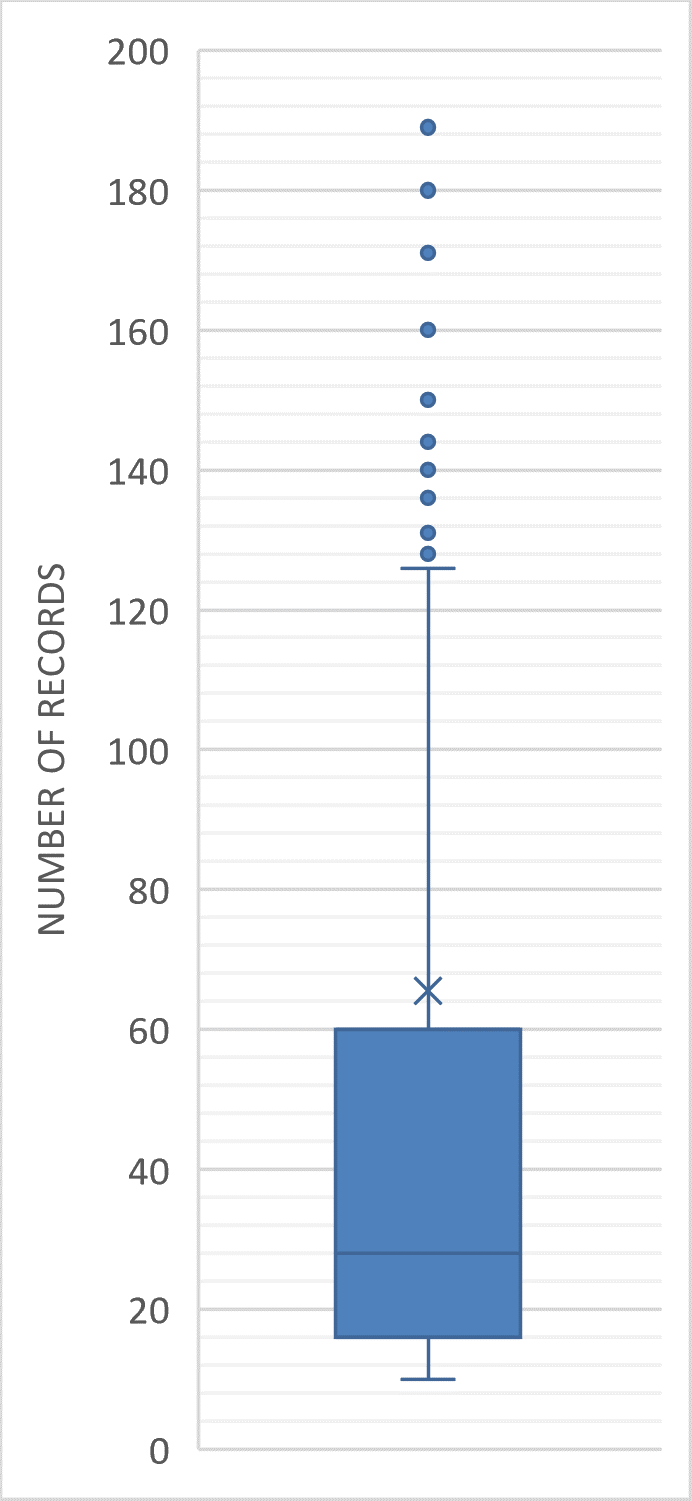


**Figure 1:** Number of records per species

## Pre-selected variables

The pre-selected variables are presented in table 10. Out of the six land use types of the land use projection, only four are retained for the SDM: Forest, Forestry, Cropland and Grassland-Pasture, with Grassland and Pasture merged due to similar habitat characteristics in Brazil (Aguiar et al., 2017; Parente et al., 2017, 2019). The land use “Other”, including urban areas and road networks, is excluded due to observation bias.

*Table 9 : Pre-selected variables in the SDMs.*

| **Variables** | | **Calibration Data** | **Future projection**  **(2015-2050)** | **Units** |
| --- | --- | --- | --- | --- |
| **Land use** | Forest | Data extracted from the MapBiomass (MapBiomas Project, 2023b; Souza et al., 2020) historical land use maps for the corresponding year of observation* | Land use projections (Silva Bezerra et al., 2022) | % |
|  | Grassland/Pasture | Data extracted from the MapBiomass (MapBiomas Project, 2023b; Souza et al., 2020) historical land use maps for the corresponding year of observation* | Land use projections (Silva Bezerra et al., 2022) | % |
|  | Cropland | Data extracted from the MapBiomass (MapBiomas Project, 2023b; Souza et al., 2020) historical land use maps for the corresponding year of observation* | Land use projections (Silva Bezerra et al., 2022) | % |
|  | Forest Plantation | Data extracted from the MapBiomas (MapBiomas Project, 2023b; Souza et al., 2020) historical land use maps for the corresponding year of observation* | Land use projections (Silva Bezerra et al., 2022) | % |
| **Topography** | Mean Slope | Slope calculated in ArcGIS based on SRTM elevation map (EROS, 2017). Average values calculated per cell at the resolution of the land use projection. | Assumed constant | ° |
|  | Mean Elevation | Averaged elevation of the SRTM (EROS, 2017) at the resolution the land use projection. | Assumed constant | m |
| **Climate** | Mean Annual Temperature | Average value of the WorldClim variable "BIO_1" over 1990-2020 aggregated at the resolution of the land use projection (Fick & Hijmans, 2017; Harris et al., 2020). | Assumed constant | °C |
|  | Mean Temperature Seasonality | Average value of the WorldClim variable "BIO_4" over 1990-2020 aggregated at the resolution of the land use projection (Fick & Hijmans, 2017; Harris et al., 2020). | Assumed constant | °C |
|  | Mean Annual Precipitation | Average value of the WorldClim variable "BIO_12" over 1990-2020 aggregated at the resolution of the land use projection (Fick & Hijmans, 2017; Harris et al., 2020). | Assumed constant | mm |
|  | Mean Precipitation Seasonality | Average value of the WorldClim variable "BIO_15" over 1990-2020 aggregated at the resolution of the land use projection (Fick & Hijmans, 2017; Harris et al., 2020). | Assumed constant | % |

** for absence data, the year 2021 is taken as reference year as it is the year where the Expert ranges maps were made.*

## Logistic model selection and calibration

After preparing the presence and absence data, a logistic model is calibrated for each species to generate a habitat suitability map for Brazil, which can then be converted into a distribution map by applying a threshold. The process is as follows: first, highly correlated variables are removed to ensure their independence. This is achieved through a collinearity test using the R function ‘*vifstep*’, which identifies and eliminates highly correlated variables (Dormann et al., 2013; Naimi et al., 2014). Variables with the highest Variance Inflation Factors (VIF) are removed iteratively until all remaining variables have a VIF inferior to 10, indicating no collinearity (Dormann et al., 2013; Naimi et al., 2014). Then, the best combination of the remaining variables is selected using the Akaike Information Criterion (AIC), a statistical measure that compares the goodness of fit across different models. The combination with the lowest AIC value is chosen as the optimal model. Although there is no consensus on the optimal variables' selection process, AIC is a reliable metric to assess calibration performances (Hooten & Hobbs, 2015; Lawson et al., 2014).The R function “*StepAIC*” is employed to identify the combination of variables that minimize the AIC (Venables & Ripley, 2002). Subsequently, the selected model is applied across Brazil to estimate the probability of species occurrence in each grid cell, indicating habitat suitability. Finally, each species’ optimal threshold for distinguishing between suitable and unsuitable habitats is determined by calculating the True Skill Statistics (TSS) for each potential threshold between 0 and 1. The threshold that maximizes the TSS is selected as the optimal value, as recommended by Jiménez-Valverde & Lobo (2007).

# References

Aguiar, D., Mello, M., Nogueira, S., Gonçalves, F., Adami, M., & Rudorff, B. (2017). MODIS Time Series to Detect Anthropogenic Interventions and Degradation Processes in Tropical Pasture. *Remote Sensing*, *9*(1), 73. https://doi.org/10.3390/rs9010073

Aiello‐Lammens, M. E., Boria, R. A., Radosavljevic, A., Vilela, B., & Anderson, R. P. (2015). spThin: An R package for spatial thinning of species occurrence records for use in ecological niche models. *Ecography*, *38*(5), 541–545. https://doi.org/10.1111/ecog.01132

Alvares, C. A., Stape, J. L., & Sentelhas, P. C. (2013). Ko¨ppen’s climate classiﬁcation map for Brazil. *Meteorol. Z.*

Barbet‐Massin, M., Jiguet, F., Albert, C. H., & Thuiller, W. (2012). Selecting pseudo‐absences for species distribution models: How, where and how many? *Methods in Ecology and Evolution*, *3*(2), 327–338. https://doi.org/10.1111/j.2041-210X.2011.00172.x

CRIA. (2024). *SpeciesLink*. https://specieslink.net/

Dormann, C. F., Elith, J., Bacher, S., Buchmann, C., Carl, G., Carré, G., Marquéz, J. R. G., Gruber, B., Lafourcade, B., Leitão, P. J., Münkemüller, T., McClean, C., Osborne, P. E., Reineking, B., Schröder, B., Skidmore, A. K., Zurell, D., & Lautenbach, S. (2013). Collinearity: A review of methods to deal with it and a simulation study evaluating their performance. *Ecography*, *36*(1), 27–46. https://doi.org/10.1111/j.1600-0587.2012.07348.x

EROS. (2017). *Shuttle Radar Topography Mission (SRTM) 1 Arc-Second Global* [Tiff]. U.S. Geological Survey. https://doi.org/10.5066/F7PR7TFT

Fick, S. E., & Hijmans, R. J. (2017). WorldClim 2: New 1‐km spatial resolution climate surfaces for global land areas. *International Journal of Climatology*, *37*(12), 4302–4315. https://doi.org/10.1002/joc.5086

Fletcher, R., & Fortin, M.-J. (2018). *Spatial Ecology and Conservation Modeling: Applications with R*. Springer International Publishing. https://doi.org/10.1007/978-3-030-01989-1

GBIF. (2024). *Global Biodiversity Information Facility*. https://www.gbif.org/

Harris, I., Osborn, T. J., Jones, P., & Lister, D. (2020). Version 4 of the CRU TS monthly high-resolution gridded multivariate climate dataset. *Scientific Data*, *7*(1), 109. https://doi.org/10.1038/s41597-020-0453-3

Hooten, M. B., & Hobbs, N. T. (2015). A guide to Bayesian model selection for ecologists. *Ecological Monographs*, *85*(1), 3–28. https://doi.org/10.1890/14-0661.1

Ibá. (2023). *Annal Report of the Brazilian Tree Industry*. https://iba.org/eng/iba-publications/annual-reports

IBGE. (2022). *Monitoramento da Cobertura e Uso da Terra*. https://www.ibge.gov.br/geociencias/informacoes-ambientais/cobertura-e-uso-da-terra/15831-cobertura-e-uso-da-terra-do-brasil.html?edicao=35138&t=o-que-e

IBGE. (2024). *Pedologia 1:2500.000*. https://www.ibge.gov.br/geociencias/informacoes-ambientais/pedologia/10871-pedologia.html?=&t=downloads

ICMBio. (2024). *Sistema de Avaliação do Risco de Extinção da Biodiversidade – SALVE*. https://salve.icmbio.gov.br/

IPCC. (2006). *2006 IPCC guidelines for national greenhouse gas inventories* (H. S. Eggleston, Buendia L., Miwa K., Ngara T., & Tanabe K., Eds.; Vol. 4). Institute for Global Environmental Strategies.

IPCC. (2019). *2019 Refinement to the 2006 IPCC Guidelines for National Greenhouse Gas Inventories* (Calvo Buendia, E., Tanabe, K., Kranjc, A., Baasansuren, J., Fukuda, M., Ngarize S., Osako, A., Pyrozhenko, Y., Shermanau, P., & Federici, S., Eds.; IPCC, Vol. 4).

Jiménez-Valverde, A., & Lobo, J. M. (2007). Threshold criteria for conversion of probability of species presence to either–or presence–absence. *Acta Oecologica*, *31*(3), 361–369. https://doi.org/10.1016/j.actao.2007.02.001

Lawson, C. R., Hodgson, J. A., Wilson, R. J., & Richards, S. A. (2014). Prevalence, thresholds and the performance of presence–absence models. *Methods in Ecology and Evolution*, *5*(1), 54–64. https://doi.org/10.1111/2041-210X.12123

MapBiomas Project. (2023a). *Annual Mapping of Soil Organic Carbon Stocks in Brazil 1985-2021 ( beta collection).* https://doi.org/10.58053/MapBiomas/DHAYLZ

MapBiomas Project. (2023b). *Collection 7.1 of the Annual Land Cover and Land Use Maps of Brazil (1985-2022)* [Dataset]. MapBiomas Data. https://doi.org/10.58053/MAPBIOMAS/VJIJCL

Marsh, C. J., Sica, Y. V., Burgin, C. J., Dorman, W. A., Anderson, R. C., del Toro Mijares, I., Vigneron, J. G., Barve, V., Dombrowik, V. L., Duong, M., Guralnick, R., Hart, J. A., Maypole, J. K., McCall, K., Ranipeta, A., Schuerkmann, A., Torselli, M. A., Lacher, T., Mittermeier, R. A., … Jetz, W. (2022). Expert range maps of global mammal distributions harmonised to three taxonomic authorities. *Journal of Biogeography*, *49*(5), 979–992. https://doi.org/10.1111/jbi.14330

MCTI. (2020). *IV Inventário—LULUCF (Uso da Terra, Mudança do Uso da Terra e Florestas, atualizado em 24/05/2021)*. https://www.gov.br/mcti/pt-br/acompanhe-o-mcti/sirene/publicacoes/relatorios-de-referencia-setorial

Michonneau, F., Brown, J. W., & Winter, D. J. (2016). rotl: An R package to interact with the Open Tree of Life data. *Methods in Ecology and Evolution*, *7*(12), 1476–1481. https://doi.org/10.1111/2041-210X.12593

Naimi, B., Hamm, N. A. S., Groen, T. A., Skidmore, A. K., & Toxopeus, A. G. (2014). Where is positional uncertainty a problem for species distribution modelling? *Ecography*, *37*(2), 191–203. https://doi.org/10.1111/j.1600-0587.2013.00205.x

Parente, L., Ferreira, L., Faria, A., Nogueira, S., Araújo, F., Teixeira, L., & Hagen, S. (2017). Monitoring the brazilian pasturelands: A new mapping approach based on the landsat 8 spectral and temporal domains. *International Journal of Applied Earth Observation and Geoinformation*, *62*, 135–143. https://doi.org/10.1016/j.jag.2017.06.003

Parente, L., Mesquita, V., Miziara, F., Baumann, L., & Ferreira, L. (2019). Assessing the pasturelands and livestock dynamics in Brazil, from 1985 to 2017: A novel approach based on high spatial resolution imagery and Google Earth Engine cloud computing. *Remote Sensing of Environment*, *232*, 111301. https://doi.org/10.1016/j.rse.2019.111301

Silva Bezerra, F. G., Von Randow, C., Assis, T. O., Bezerra, K. R. A., Tejada, G., Castro, A. A., Gomes, D. M. de P., Avancini, R., & Aguiar, A. P. (2022). New land-use change scenarios for Brazil: Refining global SSPs with a regional spatially-explicit allocation model. *PLOS ONE*, *17*(4), e0256052. https://doi.org/10.1371/journal.pone.0256052

SIRENE, MCTI, & INPE. (2020). *Dados de Uso da Terra, Mudança de Uso da Terra e Florestas*. https://www.ccst.inpe.br/cn/

Souza, C. M., Z. Shimbo, J., Rosa, M. R., Parente, L. L., A. Alencar, A., Rudorff, B. F. T., Hasenack, H., Matsumoto, M., G. Ferreira, L., Souza-Filho, P. W. M., de Oliveira, S. W., Rocha, W. F., Fonseca, A. V., Marques, C. B., Diniz, C. G., Costa, D., Monteiro, D., Rosa, E. R., Vélez-Martin, E., … Azevedo, T. (2020). Reconstructing Three Decades of Land Use and Land Cover Changes in Brazilian Biomes with Landsat Archive and Earth Engine. *Remote Sensing*, *12*(17), 2735. https://doi.org/10.3390/rs12172735

Venables, W. N., & Ripley, B. D. (2002). *Modern applied statistics with S* (4th ed). Springer.

Zizka, A., Silvestro, D., Andermann, T., Azevedo, J., Duarte Ritter, C., Edler, D., Farooq, H., Herdean, A., Ariza, M., Scharn, R., Svantesson, S., Wengström, N., Zizka, V., & Antonelli, A. (2019). CoordinateCleaner: Standardized cleaning of occurrence records from biological collection databases. *Methods in Ecology and Evolution*, *10*(5), 744–751. https://doi.org/10.1111/2041-210X.13152
